# Theranostic Imaging Agents: Integrating Molecular Diagnostics, Precision Therapeutics, and Artificial Intelligence

**DOI:** 10.3390/biom16101515

**Published:** 2026-10-18

**Authors:** Shrestha Sharma, Syed Arman Rabbani, Mohamed El-Tanani, Mohamedanas Mohamedfaruk Patni, Rakesh Kumar, Manita Saini

**Affiliations:** 1 Amity Institute of Pharmacy, Amity University, Gurugram 122413, Haryana, India; 2 Clinical Pharmacy & Pharmacology, RAK College of Pharmacy, RAK Medical and Health Sciences University, Ras Al Khaimah 11172, United Arab Emirates; 3 Department of Pharmacy, Jagannath University, Bahadurgarh 124507, Haryana, India; 4 Geeta Institute of Pharmacy, Geeta University, Naultha, Panipat 132145, Haryana, India

**Keywords:** theranostics, precision medicine, molecular imaging, radiotheranostic, multimodal imaging, artificial intelligence, personalized dosimetry, digital twins, next-generation theranostic

## Abstract

Theranostic imaging agents combine molecular diagnosis, targeted therapy and monitoring of treatment response into a single paradigm of precision medicine. This review summarizes the current development and translational landscape of theranostic imaging agents, covering molecular targets, imaging platforms, principles of agent design, nanotheranostics, radiotheranostics, and disease-specific applications. Particular emphasis is given to clinically validated and emerging targeting strategies using prostate-specific membrane antigen, somatostatin receptors, fibroblast activation protein, C-X-C Chemokine Receptor 4 (CXCR4), Human Epidermal Growth Factor Receptor 2 (HER2) and other disease-associated biomarkers. Moreover, the review addresses Positron Emission Tomography (PET), Single-Photon Emission Computed Tomography (SPECT), Magnetic Resonance Imaging (MRI), optical, photoacoustic, Raman and multimodal imaging approaches, therapeutic payloads, stimulus-responsive systems, and pharmacokinetic optimization. Moreover, emerging applications of artificial intelligence, radiomics, radiogenomics, spatial and single-cell multiomics, liquid biopsy, digital pathology, personalized dosimetry, and digital twins are evaluated for their potential to improve patient stratification, treatment selection, response assessment and adaptive therapy. Despite tremendous progress, clinical translation is hampered by target heterogeneity, off-target accumulation, radionuclide availability, manufacturing complexity, dosimetric uncertainty, regulatory requirements, limited clinical validation, data interoperability, cost and unequal access. This review, overall, emphasizes the convergence of molecular imaging, targeted therapeutics, nanotechnology, and artificial intelligence in the development of next-generation theranostic platforms, and outlines critical priorities for their clinical translation.

## 1. Introduction

The synergy between molecular imaging and therapeutic drugs has revolutionized the concept of disease management from a sequence of “diagnose then treat” to a theranostics approach, whereby a shared molecular target is used both for visualizing the disease and for its treatment. In contrast to anatomical imaging that mainly focuses on structural lesions, theranostics imaging is capable of non-invasive assessment of molecular and cellular events, thus enabling the patient selection, therapy planning, drug distribution monitoring, and therapeutic efficacy evaluation at the same time [1]. This is one of the greatest strides ever made in precision medicine because it allows treatment of patients based on their biological makeup instead of just categorizing them based on histopathology [2].

The development of theranostics has been made possible through rapid developments in fields such as molecular biology, radiochemistry, nanotechnology, bio-engineering, and advanced medical imaging [3]. The discovery of target molecules specific to the particular disease, such as prostate-specific membrane antigen (PSMA), somatostatin receptors (SSTRs), fibroblast activation protein (FAP), CXCR4, HER2, integrins, and biomarkers involved in immune response processes, has made it possible to create a pair of imaging and therapeutic agents able to detect patients who will benefit the most from the targeted therapy [4]. At the same time, progress in PET, SPECT, MRI, optical, and multimodal imaging techniques has contributed significantly to the improved characterization of disease in terms of space, time, and quantity [5].

This is especially true in oncology, where radioligand therapy has been a game changer in the treatment of metastatic prostate cancer and neuroendocrine tumors. The outstanding success recorded in the use of ^68^Ga/177Lu PSMA and ^68^Ga/177Lu DOTA-(Tyr3)-Octreotate (DOTATATE) has demonstrated that molecular imaging can not only be used for diagnosis but also as predictive biomarkers in treatment eligibility, for dosimetry optimization and for evaluation of longitudinal response [6]. Apart from these applications, several new agents for FAP, TROP-2, Nectin-4, carbonic anhydrase IX (CAIX), erythropoietin-producing hepatocellular receptor A2 (EphA2), L-type amino acid transporter 1 (LAT1), and various other pan-cancer biomarkers have entered the pipeline to extend the use of theranostics in different cancers and non-oncological diseases such as neurological disorders, cardiovascular diseases, inflammatory diseases, and infectious diseases. At the same time, the field of precision medicine is witnessing a paradigm shift towards data-based medicine [7]. AI, radiomics, radiogenomics, spatial multiomics, liquid biopsy, and digital pathology are some of the technologies that have been used along with molecular imaging for disease signature generation to enhance patient stratification, therapy selection, and outcome predictions. These technologies have turned theranostics into an ever-evolving ecosystem of clinical decision support systems that integrate imaging phenotypes along with molecular and genomic information for personalized therapies [8].

Although these are impressive developments, several issues that hinder their global translation to clinical practice still exist. These include biological heterogeneity, variability in target expression, inadequate pharmacokinetic behavior, radiation dosimetry, manufacturing challenges, accessibility to radionuclides, regulatory heterogeneity, development costs, lack of specialized infrastructure, and lack of standardization in imaging and response assessment [9]. In addition, effective clinical translation of this modality increasingly necessitates a multidisciplinary approach involving specialists from nuclear medicine, radiology, oncology, medical physics, radiochemistry, biomedical engineering, computational sciences, and regulatory science [10].

This review provides a comprehensive and forward-looking perspective on theranostic imaging agents, emphasizing the molecular principles underlying their design, emerging imaging and therapeutic platforms, nanotheranostic systems, clinically established and investigational radiotheranostics, disease-specific applications and the transformative roles of artificial intelligence and companion diagnostics. Particular attention is devoted to translational barriers, regulatory considerations, and future innovations, including smart activatable probes, digital twins, and AI-enabled adaptive theranostics that are expected to define the next generation of precision medicine and image-guided therapy.

## 2. Biological Basis of Theranostics

Theranostics operates under the premise that one particular target related to disease at the molecular level can be utilized for imaging and therapy purposes in a biological approach to “diagnose–treat–monitor” [11]. In contrast with conventional imaging, which relies more on structural changes related to the disease, molecular theranostics assesses the functional and molecular aspects of disease through abnormal receptor expression, metabolism, enzyme function, immunology and changes in the tumor microenvironment. As a result, the efficiency of the therapy provided by the theranostic agents will depend on the accuracy of the expression of these biological targets over time and space, making target validation an essential step for patient selection, personalized therapy planning, optimization of the dose used and response evaluation. New developments in genomics, proteomics, spatial transcriptomics, and single-cell sequencing have made it possible to discover new biological targets for more accurate theranostics for different disease areas [10,12].

### 2.1. Molecular Targets: Biological Basis for Theranostic Targeting

Molecular targets form the biological basis of theranostic imaging agents and are chosen depending on the following: disease specificity, higher target abundance, internalization rate, and reduced presence in non-diseased tissues [13]. The cell surface receptors are the most highly explored molecular targets, which include PSMA, SSTR2, HER2, gastrin-releasing peptide receptor (GRPR), CXCR4, integrins (α_vβ_3), and FAP. In addition to targeting receptors, novel targets include metabolic transporters (LAT1, GLUT1), hypoxic factors (CAIX, HIF-1α), immune checkpoint proteins (PD-L1, CTLA-4), angiogenic factors (VEGFR), extracellular matrix proteins, and biomarkers in the tumor stroma. Such molecular targets not only provide excellent opportunities for specific visualization but also for delivering radioactive compounds, chemotherapeutic agents, immune effectors, gene editing technologies, or light-sensitive molecules without non-specific side effects [14,15]. The range of both pan-cancer and cancer-specific targets is growing at an incredible speed, opening new perspectives for theranostics in diseases other than cancers, including neurodegeneration, heart disease, infections, and chronic inflammation.

### 2.2. Disease-Specific Biomarkers: Patient Selection and Treatment Monitoring

The disease-specific biomarkers are the molecular signatures of the pathological tissue and serve as a rationale behind the precision theranostics approach. The examples of such biomarkers for oncological conditions include PSMA, SSTR2, FAP, HER2, EGFR, CXCR4, Nectin-4 and TROP2, which assist in defining the molecular phenotype of tumors. For the neurological disorders, imaging with biomarkers such as amyloid-β, tau, α-synuclein, and translocator protein (TSPO) is becoming more common [16]. For the cardiovascular conditions, the targets for imaging include activated fibroblasts, macrophages, integrins, and matrix remodeling proteins. Biomarkers related to the activation of leukocytes, bacterial metabolism, and the migration of immune cells also get taken advantage of by inflammatory and infectious conditions. The detection of biomarkers in such conditions is being sped up more and more using multiomics approaches, spatial biology, and liquid biopsy [17].

### 2.3. Target–Biomarker Integration for Precision Theranostics

**Figure 1:**
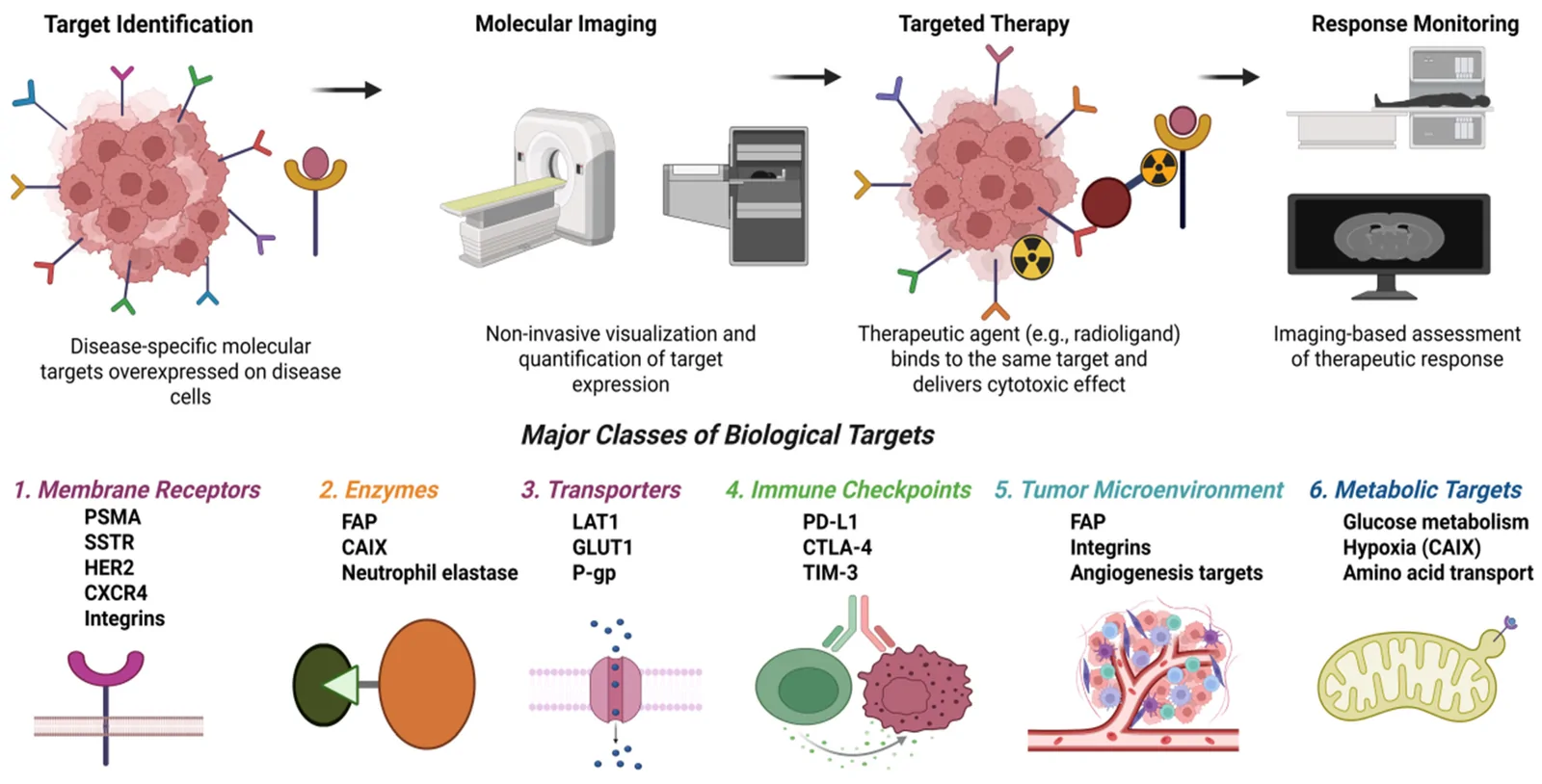
Major classes of biological targets and theranostic workflow. Created in BioRender. Rabbani, S.A. (2026) https://BioRender.com/yqnhd7v.

While there is often some overlap between these two types of biomarkers, their purposes are different but complementary in the context of theranostics. Specifically, imaging biomarkers assess biological processes using non-invasive imaging techniques that deliver information about the expression of targets, extent of disease, pharmacokinetics, biodistribution, occupancy of receptors, and early treatment response using quantitative techniques like PET, SPECT, MRI, and hybrid imaging. On the other hand, therapeutic biomarkers assess the patient population that would benefit the most from the intervention using a particular technique [15,18]. In modern theranostics, these functional groups become more convergent in the form of companion biomarkers, where diagnostic imaging proves the availability of the target before administration of therapy and simultaneously serves to monitor the efficacy of treatment using the same molecular entity [19]. It is through such a biomarker system that precision medicine acquires its unique characteristic of integrating imaging into therapy decisions and management (Figure 1).

## 3. Molecular Imaging Platforms

Molecular imaging provides the biological and quantitative basis for theranostics, allowing non-invasive characterization of disease-specific targets, tissue function, tracer distribution and response to therapy. Rather than viewing PET, SPECT, MRI, optical imaging, ultrasound and photoacoustic imaging as competing technologies, their value should be assessed based on the biological information they provide, their quantitative capability, spatial resolution, tissue penetration and compatibility with therapeutic intervention [20,21,22].

PET and SPECT are the most clinically relevant of the available methods for molecular theranostics, particularly because radiolabeled probes can provide whole-body target visualization and quantitative information to directly support patient selection and radiopharmaceutical therapy. PET has high sensitivity and quantitative capability, while SPECT has broader radionuclide compatibility and is still particularly relevant for post-therapy imaging and dosimetric assessment. Recent progress in targeted radiotracers is pushing molecular imaging beyond conventional metabolic evaluation to the characterization of disease-specific targets, tumor heterogeneity, and treatment response. However, quantitative accuracy is still affected by tracer kinetics, acquisition and reconstruction parameters, scanner calibration, lesion size and biological heterogeneity. Hence, the clinical value of molecular imaging depends not only on the visualization of the targets but, more and more, on the reproducibility and decision relevance of the quantitative information achieved [22,23,24,25,26,27].

MRI, optical, ultrasound, photoacoustic, and Raman-based approaches provide complementary information to improve anatomical localization, tissue characterization, functional assessment, or real-time guidance. Thus, their major contribution to theranostics is complementary and not necessarily competitive with nuclear imaging. MRI provides high soft tissue contrast and multiparametric characterization without ionizing radiation, optical and Raman approaches provide high molecular specificity but are generally limited by tissue penetration, ultrasound provides real-time imaging and opportunities for image-guided delivery, and photoacoustic imaging combines optical contrast with ultrasonic detection and is being explored for functional and molecular imaging. Many of these approaches remain in the preclinical or specific application stage, and their wider clinical impact will depend on probe safety, reproducibility, penetration depth, standardization, and demonstration of incremental clinical benefit [25,26,27,28,29,30].

Thus, the emerging direction is multimodal rather than modality-specific imaging. New multimodal probes integrate nuclear, magnetic, optical, or photoacoustic signals into a single platform and hybrid systems like PET/CT, PET/MRI and SPECT/CT combine molecular information with anatomical localization. This combination can improve lesion localization, biodistribution assessment, treatment monitoring and image-guided intervention, but added complexity does not always translate into higher clinical utility. For example, recent work on multimodal SPECT probes highlights the potential of integrating complementary imaging signals as well as the ongoing chemical, biological, and translational barriers to clinical implementation [23,24,25,26,27,28,30,31,32,33,34].

Therefore, the future development of molecular imaging in theranostics should be transiting from the addition of more modalities to quantitative, standardized, and clinically actionable imaging. Clinical value is increasingly determined by harmonized acquisition and reconstruction, reliable image quantification, longitudinal assessment, multimodal data integration, and appropriate validation [35,36,37,38]. AI could further aid this transition through support of image reconstruction, segmentation, quantitative analysis, multimodal fusion, and response assessment; however, the ultimate measure of computational improvement should be whether it improves patient stratification, dosimetry, treatment monitoring, or clinical decision-making rather than merely technical performance [39]. The main features, typical applications, benefits and drawbacks of these platforms are summarized in Table 1.

## 4. Design Principles of Theranostic Imaging Agents

Theranostic imaging agents are designed on the basis of molecular recognition, imaging function, therapeutic activity, and advantageous pharmacokinetics in a combined multifunctional structure. An ideal theranostic agent should have excellent target specificity, quick and stable target localization, low non-specific binding, high signal-to-background ratio, effective therapeutic delivery, easy excretion, and good biocompatibility [48]. Therefore, the latest generation of theranostics consists of five interconnected elements: (i) targeting ligands, (ii) imaging units, (iii) therapeutic cargoes, (iv) stimulus response, and (v) pharmacokinetic optimization. The synergistic integration of these elements determines imaging sensitivity, therapeutic index, safety profile and ultimately clinical success in precision medicine [49].

### 4.1. Targeting Ligands

Targeting ligands contribute to the specificity of drug carriers by selective recognition of disease-specific biomarkers at high affinity and minimal association with healthy tissues. Ligand selection is dictated by the accessibility of the target, receptor density, uptake kinetics, immunogenicity, and biological stability. Monoclonal antibodies and fragments thereof have excellent specificity but long plasma half-life and poor tissue permeability, while peptides, small molecules, nanobodies, aptamers, affibodies, and engineered proteins enable fast tumor entry and good pharmacokinetics [15,16,17,18]. Ligands that have been clinically proven to bind to PSMA, SSTR2, HER2, CXCR4, GRPR, FAP, and integrins have made molecular targeting an essential part of theranostics today. New methods for engineering ligands that involve multivalent structures, bi-specific ligands, and biomimicry targeting have been developed to improve selectivity while dealing with biological variability and resistance [20,21].

### 4.2. Imaging Moieties

Imaging moieties translate molecular recognition into a measurable signal for diagnostics and define the sensitivity, resolution, and clinical use of theranostics. Radionuclides still dominate as imaging modalities, where positron-emitting radionuclides such as 18F, ^68^Ga, ^64^Cu, and ^89^Zr are used for PET imaging, and gamma-emitting radionuclides like ^99^mTc and ^111^In are used for SPECT imaging [21,22,23]. MRI contrast agents consisting of gadolinium, iron oxide nanoparticles, and manganese allow more precise anatomical resolution, while fluorescent dyes, NIR-I/NIR-II dyes, photoacoustic chromophores, and SERS nanoparticles enable high-resolution optical imaging [24,25]. The current trend is toward multimodal imaging probes capable of simultaneously integrating PET, MRI, optical, photoacoustic or Raman imaging, thereby providing complementary molecular, anatomical, and functional information from a single agent.

### 4.3. Therapeutic Payloads

Therapeutic cargoes serve as the effector moiety in a theranostic molecule that is chosen based on the biology of the disease, intracellular trafficking, and the mode of action of the therapy. The radiotherapeutic isotopes ^177^Lu, ^225^Ac, ^90^Y, and ^161^Tb have transformed targeted radionuclide therapy by providing localized cytotoxic radiation without harming the neighboring healthy cells [50]. In addition to radiotherapy, other therapeutics such as chemotherapy, photodynamic therapy, photothermal therapy, immune therapy, gene editing, siRNA, mRNA, Clustered Regularly Interspaced Short Palindromic Repeats (CRISPR-Cas systems), and protein therapeutics have become a part of the theranostic molecules. The emergence of multifunctional nanotheranostic platforms capable of co-delivering multiple therapeutic modalities further enables synergistic treatment and adaptive precision therapy [51,52].

### 4.4. Stimulus-Responsive Systems

Stimulus-responsive theranostic systems utilize the concept of stimulus-dependent activation by harnessing physicochemical or biological features of diseased states. Endogenous stimuli such as acidity, reactive oxygen species (ROS), hypoxia, glutathione, overexpression of enzymes, and redox imbalance trigger selective activation in diseased regions, while exogenous stimuli like light, ultrasound, magnetic field, and ionizing radiation allow external regulation for therapeutic delivery [53]. The integration of activatable probes, self-reporting nanocarriers, and feedback-controlled imaging systems represents a major advancement toward intelligent and adaptive theranostic platforms.

### 4.5. Pharmacokinetic Optimization

Pharmacokinetics optimization is critical in maximizing target uptake and minimizing exposure and toxicities to normal tissues. Rational engineering of these agents involves optimizing the molecule’s molecular weight, surface charge, hydrophilicity, ligand density, linker, plasma stability, and degradation in order to enhance circulation time, target penetration, cellular uptake, and background clearance [54]. Surface modification approaches including PEGylation, zwitterionic surfaces, albumin binding, biomimetic membrane shielding, and stealth nanoparticles increase in vivo stability. Artificial intelligence-aided drug design and pharmacokinetics simulation, as well as quantitative imaging-based dosimetry, are now more and more often included in the theranostics development process, allowing for the prediction of optimal biodistribution and efficacy [55]. Design principles and components of theranostic imaging agents are depicted in Table 2.

## 5. Nanotheranostic Platforms: Design Strategies and Therapeutic Applications

Nanotheranostic platforms integrate therapeutic delivery with diagnostic or imaging functionality within a nanoscale system, enabling treatment localization, biodistribution assessment, and, potentially, real-time monitoring of therapeutic response. Their value depends not simply on the presence of an imaging component, but on the extent to which the physicochemical properties of the nanoplatform determine cargo loading, biological distribution, target interaction, imaging signal generation, and therapeutic activity. Accordingly, the principal nanotheranostic platforms can be distinguished by the mechanisms through which they incorporate or generate imaging signals and couple these signals with therapy [51,53].

### 5.1. Liposomes

Liposomes are the most advanced nanotheranostic system due to their high biocompatibility, biodegradability, and dual loading capacity of hydrophilic and hydrophobic drugs. Functionalizing their surface with antibodies, peptides, aptamers, or other ligands allows for precise molecular targeting, while loading them with radionuclides, Magnetic Resonance Imaging contrast agents, fluorescent molecules, or photoacoustic imaging agents allows for multimodal imaging [66]. Advanced stealth and hybrid liposome formulations have combined chemotherapy, radiotherapy, immunotherapy, or gene editing with imaging in order to achieve theranostics. Liposomal theranostics have demonstrated significant translational potential by improving drug bioavailability, overcoming multidrug resistance, and minimizing off-target toxicity [67].

### 5.2. Polymeric Nanoparticles

Polymeric nanoparticles are versatile nanocarriers for precise theranostics due to their modifiable structures and high versatility. Biodegradable polymeric materials such as Poly(lactic-co-glycolic acid) (PLGA), Polyethylene Glycol (PEG), chitosan, polycaprolactone and dendrimers have been utilized to control drug delivery, enhance their systemic retention and provide tailored degradation behavior [68]. It provides a versatile platform for the incorporation of therapeutic molecules and imaging agents through encapsulation, covalent conjugation, or surface functionalization. Imaging can be achieved by incorporation of fluorophores for fluorescence or near-infrared imaging, radionuclides for PET or SPECT, or suitable contrast-generating components for MRI and other modalities. The tunable polymer composition, particle size, surface chemistry and degradation behavior can influence circulation, cellular uptake, drug release and imaging performance. The theranostic benefit is therefore the sum of controlled delivery of therapeutics and spatial information on nanoparticle distribution or treatment response. However, the additional imaging functionality may contribute to complexity in formulation and may modify the physicochemical properties, pharmacokinetics, and reproducibility of manufacture. Recent reviews highlight that the main challenge is not to increase the multifunctionality of the nanoparticles, but to build predictable structure–function relationships [54,69].

### 5.3. Dendrimers and Micelles

Dendrimers and polymeric micelles are very well-engineered examples of nanosystems, showing excellent uniformity and multifunctionality at molecular scale. Dendrimers are nanocarriers with designed branched structures and multiple surface functional groups, allowing high drug loading, multivalency targeting and co-encapsulation of imaging and therapeutic molecules [66]. They exhibit highly branched architectures that are monodisperse and contain a large number of terminal functional groups that permit the incorporation of therapeutic and imaging components into internal cavities or onto the surface. Imaging agents may be fluorescent molecules, radionuclides, iodine-containing agents for CT or paramagnetic species for MRI. Their multivalency also enables simultaneous conjugation of targeting ligands and therapeutic molecules, resulting in a compact platform where molecular recognition, imaging and treatment can be combined. However, increased generation or surface functionalization can alter toxicity, clearance, protein interactions and biodistribution. Thus, the imaging advantage of dendrimers should be considered in light of their pharmacokinetics and safety implications, rather than as a stand-alone advantage [70].

Polymeric micelles are self-assembled structures of amphiphilic molecules with a hydrophobic core and hydrophilic corona and are especially suitable for solubilizing poorly water soluble therapeutic agents. The theranostic role can be achieved via the use of fluorescent or near-infrared probes, radionuclides, magnetic contrast elements or stimulus-responsive imaging elements in the micellar structure. This enables imaging of micelle distribution or target accumulation, while the same carrier delivers the therapeutic payload. Stimulus-responsive micelles can also link imaging or activation to tumor-associated conditions such as pH, enzymes, hypoxia, heat, light, magnetic fields or ultrasound. However, micelle stability, premature disassembly, interactions with proteins and differences in behavior between in vitro and in vivo are still important limitations [69,70].

### 5.4. Exosomes and Biomimetic Nanoparticles

Exosomes and biomimetic nanoparticles utilize the properties of biological membranes to enhance cellular interaction, targeting, and delivery. Fluorescent probes, membrane-bound contrast agents, radionuclide labeling, or other imaging reporters can impart imaging potential to them, allowing investigation of their biodistribution, cellular uptake and therapeutic delivery. The biological membrane can be used as targeting and delivery interface, which can potentially overcome some limitations of the conventional synthetic carriers. But heterogeneity in biological source, isolation and purification, cargo loading, characterization, storage and batch-to-batch reproducibility continue to be a major challenge. For biomimetic systems, the key translational question is whether the biological features being mimicked translate into reproducible improvements in delivery and imaging under clinically relevant conditions [71,72].

### 5.5. Metal Nanoparticles

Metal–organic frameworks (MOFs) are porous crystalline materials made of metal nodes or clusters coordinated to organic linkers. Their huge surface area, tunable pore architecture and chemically modifiable composition provide opportunities to incorporate therapeutic molecules and imaging components in the same platform. Importantly, the imaging functionality may be derived from the intrinsic properties of the metal centers or from incorporated probes [66,67,68]. Based on their composition and design, MOFs have been investigated for MRI, CT, fluorescence, photoacoustic, PET, SPECT and multimodal imaging. Their open architecture allows the simultaneous loading and controlled release of drugs. Stimulus-responsive degradation or tumor-microenvironment activation can connect imaging with therapeutic activity. Recent studies show the versatility of MOFs but also raise biodegradation, pharmacokinetics, toxicity, manufacturing and long-term biosafety as major barriers to translation [54,69].

**Figure 2:**
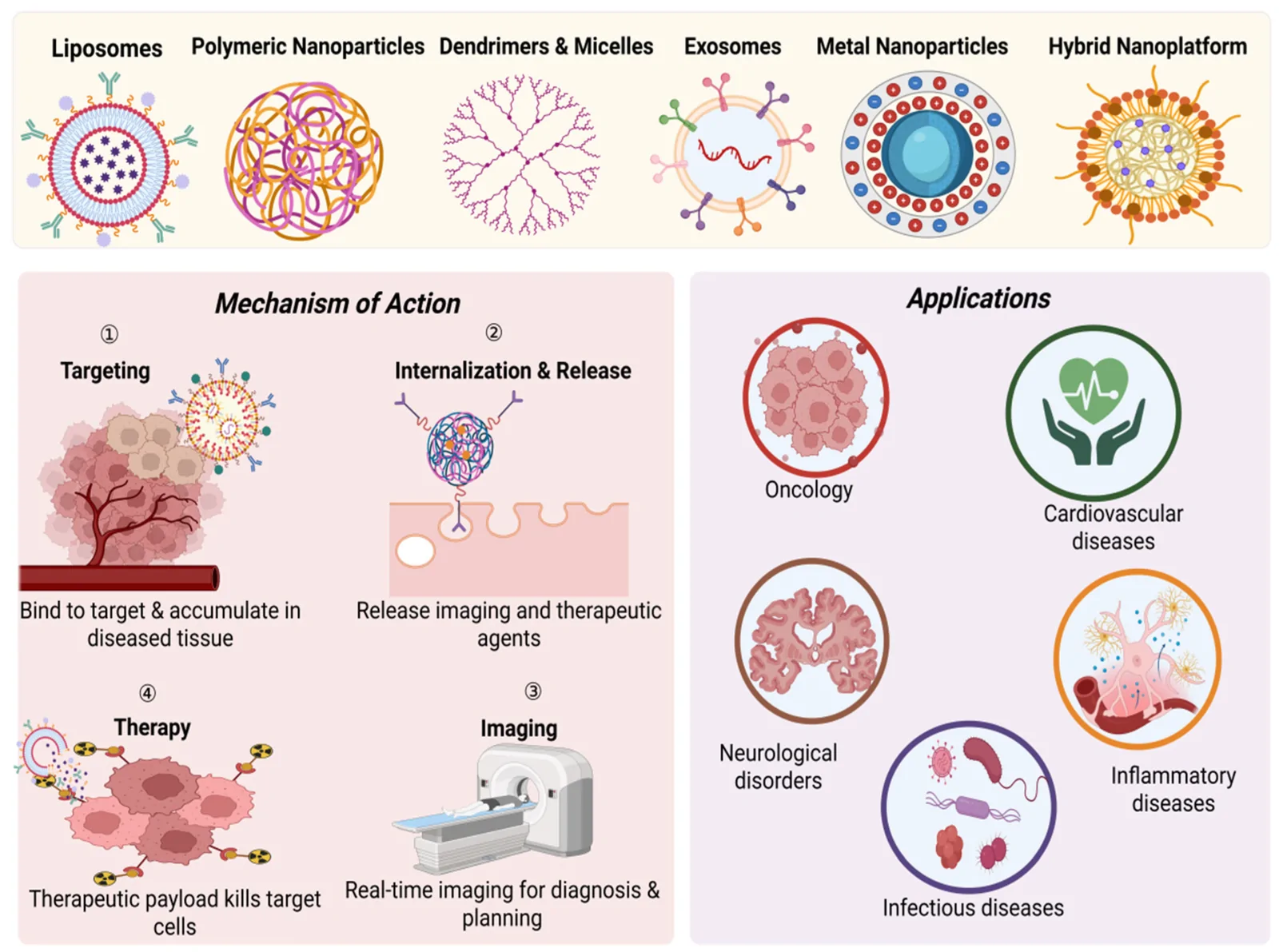
Nanotheranostics with mechanism and their applications. Created in BioRender. Rabbani, S.A. (2026) https://BioRender.com/ie8a1mj.

The latest developments in the design of bioinspired MOFs along with nanohybrids based on lipids, polymers, inorganic nanomaterials, and biomembranes have helped solve the problems associated with stability, biocompatibility, and immune responses and, at the same time, enhanced delivery control, tumor targeting, and clinical translation [73]. These intelligent hybrids are considered the next generation of nanotheranostics that will be able to perform diagnostics and therapy monitoring in one system (Figure 2).

### 5.6. Critical Perspective

The basic scientific idea across all these platforms is the integration of structure, imaging mechanism, biodistribution and therapeutic function, not just the integration of multiple functions into one nanoparticle. Imaging components can report on nanoparticle localization, target engagement, drug release, physiological response or therapeutic outcome, but these signals are not necessarily interchangeable. A detectable imaging signal does not automatically prove therapeutic delivery, nor does nanoparticle accumulation automatically prove effective target engagement. Therefore, the evaluation of nanotheranostic platforms should be based on the correlation of imaging readouts with clinically relevant therapeutic endpoints. Recent advances emphasize a growing focus on multimodal and stimulus-responsive systems. Translation will depend on reproducible manufacturing, predictable pharmacokinetics, validated imaging–therapy relationships, and the demonstration of benefit beyond established therapeutic approaches.

## 6. Radiotheranostics: The Clinical Revolution

Radiotheranostics can be considered to be the most developed clinical application of precision medicine due to its utilization of molecular imaging and radionuclide therapy via the use of radiopharmaceuticals that are chemically identical or highly similar [74]. This approach allows for non-invasive target localization, patient selection, dosimetry, drug delivery, and response assessment using a single molecular tool. The FDA approval of [^177^Lu] Lu-DOTATATE (Lutathera^®^, Novartis Pharma AG, Basel, Switzerland) and [^177^Lu] Lu-PSMA-617 (Pluvicto^®^, Novartis Pharma AG, Basel, Switzerland) has changed the paradigm of radiotheranostics from a research tool to a standard treatment method, which has led to the creation of many next-generation radioligands that target cancer cells and tumor microenvironment [75]. The current clinical landscape is rapidly expanding from receptor-specific therapies toward adaptive, biomarker-driven radiopharmaceutical platforms capable of integrating molecular imaging, dosimetry, artificial intelligence, and combination therapies.

### 6.1. PET-Guided Radionuclide Therapy

PET imaging is the foundation of modern radiotheranostics, through quantitative measurement of target expression prior to treatment, determination of radiation dosage, and measurement of response to treatment. PET imaging agents that utilize positron-emitting radionuclides (such as ^68^Ga, ^18^F, ^64^Cu, and ^89^Zr) select patients who can potentially respond to targeted radionuclide therapy, whereas therapy radionuclides like ^177^Lu, ^225^Ac, ^90^Y, and ^161^Tb deliver cytotoxic radiation targeting the same molecular target [21,22,23]. This imaging-guided treatment paradigm enables individualized dosimetry, optimization of treatment cycles, early response assessment, and adaptive therapeutic decision-making, establishing PET as an indispensable companion diagnostic for precision oncology [24].

### 6.2. PSMA

PSMA-targeted radiotheranostics serve as the gold standard of successful clinical application in targeted radionuclide therapy. Overexpressed in metastatic castration-resistant prostate cancer, PSMA allows for precise staging of the disease through the use of ^68^Ga-PSMA or ^18^F-PSMA PET/CT imaging, followed by treatment via ^177^Lu-PSMA-617 or α-particle emitting ^225^Ac-PSMA radiotracers. Apart from contributing to improved progression-free and overall survival, PSMA theranostics also allows for lesion-specific dosimetry, evaluation of intra-patient heterogeneity, and adaptive treatment planning [28,29,30]. The future of PSMA theranostics includes research into new PSMA ligands, radiohybrids and albumin.

### 6.3. Somatostatin Receptor

SSTR targeting for theranostics has completely transformed the way we manage gastroenteropancreatic neuroendocrine tumors (GEP NETs). PET scanning with ^68^Ga-DOTATATE, DOTA-(Tyr3)-Octreotide (DOTATOC), or DOTA-1-Nal3-octreotide (DOTANOC) helps in accurate diagnosis of SSTR-positive tumors and facilitates peptide receptor radionuclide therapy (PRRT) with ^177^Lu-DOTATATE that has been found to provide sustainable clinical outcomes with a favorable safety profile [31]. Recent advancements involve alpha-emitter-based PRRT, radioligand antagonists with better tumor uptake, personalized dosimetry, and combination therapies with radio sensitizers and immunotherapies to surmount.

### 6.4. FAPI

Fibroblast activation protein (FAP), mainly found on cancer-associated fibroblasts in tumor stroma, has quickly established itself as one of the most exciting pan-cancer theranostic targets. FAPI PET/CT exhibits an excellent tumor-to-background ratio in many types of cancers, such as pancreatic, breast, colorectal, lung, and hepatobiliary cancers, sometimes even superior to ^18^F-FDG PET. The therapeutic radiopharmaceuticals with ^177^Lu, ^90Y^, ^225^Ac, and ^161^Tb have entered clinical trials and are a means to attack both cancer cells and tumor stroma [76,77]. The broad applicability of FAP-targeted radiotheranostics positions it as a leading candidate for next-generation precision oncology.

### 6.5. CXCR4

CXCR4 chemokine receptor is often upregulated in various forms of hematological cancer as well as advanced solid cancers, playing a role in tumor growth, metastasis, angiogenesis, and resistance to treatment. Radiotheranostics compounds like ^68^Ga-pentixafor for PET visualization and ^177^Lu/^90^Y-pentixather for targeted radiotherapy show promising clinical efficacy, especially in multiple myeloma, acute leukemia, lymphomas, and some highly aggressive solid cancers [78]. Given the fact that CXCR4 expression is associated with aggressiveness and metastatic potential of tumors, CXCR4-targeted radiotheranostics provides unique possibilities for risk stratification, personalized medicine, and immunotherapy combinations [79].

### 6.6. HER2

HER2-based radiotheranostics builds upon the concept of molecular imaging in the field of antibody therapy in terms of assessing quantitatively the amount of receptor expression and targeting of the radionuclide. Radioactive tracers that have been radiolabeled with ^89^Zr, ^64^Cu, or ^68^Ga allow whole-body assessment of HER2 heterogeneity and identification of patients eligible for HER2-based therapies. Simultaneous development of HER2-based therapeutics using ^177^Lu, ^225^Ac, and other therapeutic isotopes appears to be an interesting approach in the treatment of HER2-positive breast, stomach, ovarian, and other HER2 expressing tumors [76,77].

### 6.7. Integrin-Targeted Systems

Integrins such as αvβ3 and αvβ6 are responsible for the process of tumor angiogenesis, invasion, remolding of the extracellular matrix, and metastasis dissemination. Hence, integrins form an interesting target for radionuclide therapy and molecular imaging. RGD-based radioligands conjugated with ^68^Ga, ^18^F, or ^64^Cu offer highly sensitive detection of angiogenesis. ^177^Lu and ^90^Y are under study as the therapeutic isotopes for selective radiation targeting of angiogenic vasculature within tumors [78]. Integrin-based radiotheranostic agents will work more effectively in combination with antiangiogenic agents, immune checkpoint inhibitors, and external beam radiotherapy.

### 6.8. Emerging Radionuclides

The future of the field of radiotheranostics lies in the development of novel radionuclides that are better optimized in terms of their physical properties and radiobiological effects. Although the most prominent clinical radionuclide is still ^177^Lu, growing interest is shown in alpha emitters ^225^Ac, ^212^Pb, and ^227^Th, which provide radiation with high-linear energy transfer to overcome radioresistance with minimum harm. At the same time, new beta emitters like ^161^Tb, ^67^Cu, and ^47^Sc along with appropriate diagnostic radionuclides like ^44^Sc, ^64^Cu, and ^89^Zr allow for true isotopic theranostics [79]. Breakthroughs in the fields of radiohybrid chemistry, chelator design, isotope production, and AI-based dosimetry models will undoubtedly speed up the progress towards individualized radiopharmaceutical treatment, thus positioning radiotheranostics as one of the key pillars of future precision oncology. Clinically approved and investigational theranostic imaging agents are shown in Table 3.

## 7. Disease-Specific Clinical Applications

**Figure 3:**
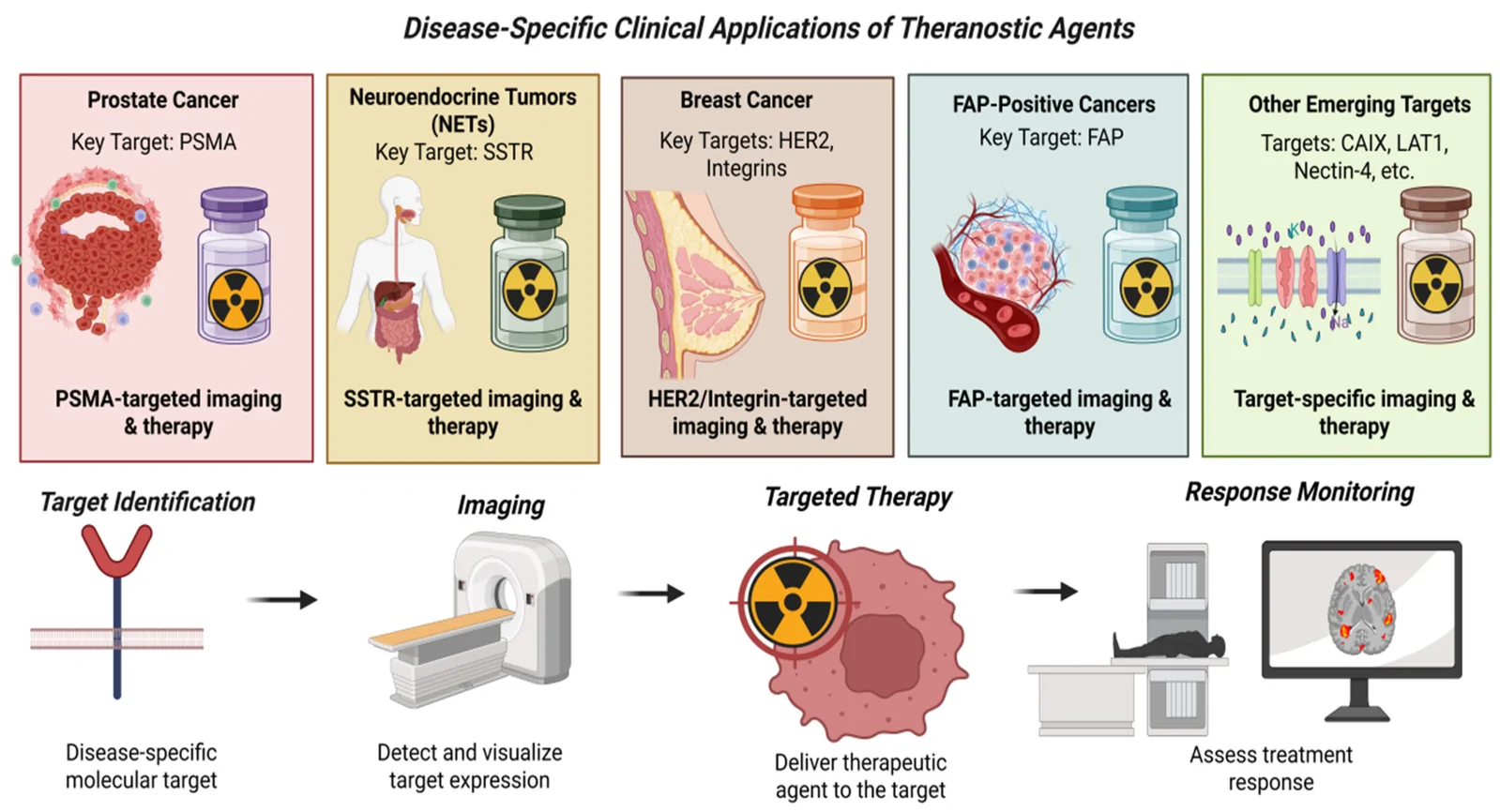
Clinical application of theranostic agents in different disease conditions. Created in BioRender. Rabbani, S.A. (2026) https://BioRender.com/b4d2e8t.

The translation of theranostic imaging from proof-of-concept into the realm of disease-specific precision medicine is characterized by the use of molecular imaging for target identification along with tailoring therapy to the individual, predicting response to treatment, providing quantitative dosimetry, and allowing disease monitoring over time. Although oncology continues to be the primary area of theranostics because of its success in radionuclide therapy, theranostics is quickly becoming applicable in neurology, cardiology, infections, and autoimmune diseases [104]. This evolution reflects a paradigm shift from organ-based diagnosis to molecular phenotype-driven patient management, where imaging biomarkers function as companion diagnostics for targeted therapeutic intervention, as shown in Figure 3.

### 7.1. Oncology

Oncology is considered the most advanced field where theranostics have been used extensively, owing to the presence of specific molecular targets within tumors and matched diagnostic–therapeutic radiopharmaceuticals. Modern theranostics for oncology makes use of PET/SPECT imaging along with radionuclide therapy, nanomedicine, antibody drug conjugates, and immunotherapy in order to facilitate patient stratification, dosimetric tailoring, adaptive planning, and real-time monitoring of therapy [97,105]. Apart from existing molecular targets, including PSMA and somatostatin receptors, new biomarkers like FAP, HER2, CXCR4, integrins, Nectin-4, TROP2, and carbonic anhydrase IX are widening the scope of precision medicine in oncology.

#### 7.1.1. Prostate Cancer

Prostate cancer can be considered a typical example of radiotheranostic approaches in medicine. PSMA PET/CT is currently the gold standard diagnostic method for staging, biochemical recurrence, and therapeutic decision-making, whereas ^177^Lu-PSMA-617 and novel ^225^Ac-PSMA treatment approaches have proven to provide significant progress in progression-free survival and quality of life in metastatic castration-resistant prostate cancer patients [89,90,91]. Currently, efforts are directed at developing radiohybrid ligands, alpha-emitter therapy, combinations with AR-inhibitors and immunotherapy, and AI-aided dosimetry.

#### 7.1.2. Breast Cancer

The field of breast cancer theranostics has progressed from simple HER2 detection to advanced molecular profiling through HER2-specific PET scanning and radioligand therapy. Advanced radiopharmaceutical drugs specifically designed for HER2, ERs, FAP, and immune-associated biomarkers allow for non-invasive analysis of receptor heterogeneity, resistance to treatment, and metastasis [106]. Coupling with ADCs and antibody radiolabeling is leading to individualized cancer therapies, especially those involving HER2-positive and triple-negative breast cancers.

#### 7.1.3. Lung Cancer

In lung cancer theranostics, there is an increasing use of molecular targets such as EGFR, HER2, FAP, CXCR4, PD-L1, and integrins. Assessment of the expression of the receptors using PET has enabled personalized targeted treatments, and radiopharmaceuticals coupled with immunotherapy and kinase inhibitors present hopeful ways of overcoming the heterogeneity and drug resistance of tumors [107].

#### 7.1.4. Glioblastoma

Glioblastoma is among the most difficult malignancies owing to significant intratumoral heterogeneity and the blood–brain barrier. Theranostics imaging involving targeting amino acid transporters, integrins, EGFR, CXCR4, and TSPO has an advantage over regular MRI in terms of better delineation, planning therapy, and detecting recurrences. Nanotheranostic systems that can cross the blood–brain barrier and carry radionuclides, chemotherapy, and gene editing systems seem promising [108].

#### 7.1.5. Neuroendocrine Tumors

Neuroendocrine tumors are an example of clinical validation of peptide receptor radionuclide therapy (PRRT). ^68^Ga-DOTATATE/DOTATOC PET imaging helps in the identification of somatostatin receptor expression and administration of treatment using ^177^Lu-DOTATATE for disease control and prolongation of survival. Present studies focus on the development of radioligands based on antagonists, the use of alpha emitting radionuclides, and personalized dosimetry [109].

#### 7.1.6. Melanoma

Theranostic methods for the management of melanomas have become increasingly melanin-, integrin-, FAP- and immune checkpoint-based with regard to the characterization of the disease and therapy planning. Molecular imaging can be used to diagnose metastatic disease and quantify immunotherapy response, whereas radioactive drugs and photoablation therapies using nanoparticles provide complementary therapeutic options for late-stage disease [101].

#### 7.1.7. Pancreatic Cancer

Pancreatic Ductal Adenocarcinoma (PDAC) still represents an area of high potential for development due to its rich tumor stroma environment and poor prognosis. FAP-based PET imaging, targeting probes for integrins, mesothelin-based therapies, and stromal nanotheranostics are all shown to provide better visualization and effective delivery of drugs to tumors. The multifunctional nanoparticle therapy that combines chemotherapy, immunotherapy, and imaging is being studied more and more intensively [107,108,109].

### 7.2. Neurology

Theranostics for neurological disorders is becoming increasingly relevant and is currently moving beyond the scope of diagnosis through molecular imaging to targeting diseases. The combination of PET imaging with disease-modifying treatments, nanomedicine, and the use of drugs that pass through the blood–brain barrier is helping achieve early diagnosis and patient treatment [110].

#### 7.2.1. Alzheimer

Alzheimer’s disease theranostics imaging includes amyloid-β, tau and neuroinflammation. The recent development of PET agents directed against amyloid and tau is used as a companion diagnostic for anti-amyloid monoclonal antibody therapy, and the development of new theranostic systems based on nanoparticles is trying to integrate disease diagnosis and delivery of drugs through the blood–brain barrier [110].

#### 7.2.2. Parkinson Disease

In Parkinson’s disease, molecular imaging of dopaminergic transporters, α-synuclein protein aggregation, mitochondrial dysfunction, and neuroinflammation is useful for early diagnosis and treatment of the disease. Experimental theranostic systems using neuroprotective nanoparticles, gene therapy, and molecular imaging have been proposed to manage disease progression.

#### 7.2.3. Glioma

Theranostics for glioma combines amino acid PET imaging, MRI, and nanomedicine to facilitate accurate delineation of the tumor, molecular profiling, and therapy follow-up. Novel radiotheranostics and biomimetic nanoparticles that can cross the blood–brain barrier allow the use of image-guided personalized therapy in malignant gliomas [108,109].

### 7.3. Cardiovascular Diseases

Theranostic approaches in cardiovascular medicine are shifting from anatomy-based imaging to molecular profiling of inflammation, fibrosis, angiogenesis, and vascular remodeling. More frequently, targeted imaging probes are employed to detect at-risk individuals and initiate treatment on a personalized basis prior to any structural changes becoming irreversible [111,112].

#### 7.3.1. Vulnerable Plaque

Molecular imaging of vulnerable atherosclerotic lesions is achieved by targeting activated macrophages, vascular inflammation, integrins, oxidized low-density lipoproteins, and fibroblast activation protein in order to detect high-risk lesions which may suffer from plaque rupture. The use of theranostic nanomaterials for delivering anti-inflammatory drugs, statins, or RNA therapies could allow both plaque detection and stabilization, preventing myocardial infarction and strokes [113,114].

#### 7.3.2. Myocardial Fibrosis

Imaging targeted to fibroblast activation protein is a very promising strategy for assessing myocardial remodeling and fibrosis after ischemic damage or cardiomyopathy. The combination of molecular imaging with anti-fibrotic therapies can be used to enable early diagnosis, treatment, and follow-up of cardiac remodeling without the risk of functional impairment [93,94].

### 7.4. Infectious Diseases

The application of theranostic imaging to infectious diseases can now be achieved via the synthesis of pathogen-targeted and host-oriented molecular imaging probes that allow the distinction between infection and sterile inflammation [115]. This is made possible by the use of radiolabeled antimicrobial peptides, bacterium-specific metabolic substrates, and pathogen-targeted nanocarriers that allow rapid identification of pathogens, evaluation of therapy efficacy, and guided antimicrobial delivery with reduced systemic toxicity [116].

### 7.5. Autoimmune Diseases

Autoimmune diseases offer great potential for theranostic application since their activity involves clearly identifiable markers of inflammation and immune cells. Molecular imaging of activated macrophages, fibroblast activation protein, integrins, cytokine receptors, and immune cell trafficking provides an early detection method, disease activity assessment, and individual therapy management. The novel nanotheranostic technology that can deliver biologics, immunomodulatory small molecules, nucleic acids, and genetic editing drugs while also enabling treatment visualization is anticipated to revolutionize the treatment of rheumatoid arthritis, inflammatory bowel disease, systemic lupus erythematosus, and multiple sclerosis [93,101]. Disease-specific theranostic applications are summarized in Table 4.

## 8. AI-Enabled Theranostics: From Image Analysis to Adaptive Treatment

Artificial intelligence (AI) is transforming theranostics from static image reading to quantitative, predictive and adaptive treatment systems. Its main value is not just automation, but the fusion of molecular imaging with clinical, pathological, genomic and longitudinal data to solve biological heterogeneity and guide personalized therapeutic choices [119,120]. Present applications include lesion segmentation, molecular target quantification, patient selection, radiomics, response prediction and personalized dosimetry, while new models integrate multimodal learning and theranostic digital twins [121,122].

### 8.1. AI for Diagnostic Imaging and Tumor Characterization

Radiomics and deep learning have exponentially expanded the quantitative capabilities of diagnostic imaging. Radiomics extracts high-dimensional features related to tumor intensity, texture, morphology, spatial heterogeneity, and temporal variations. Deep learning models can learn imaging representations directly for lesion detection, segmentation, classification, and quantitative analysis. These approaches have been used in PET, SPECT, CT and MRI for tumor characterization, molecular phenotype prediction, disease classification and treatment response assessment [123,124,125,126,127]. Their relevance to theranostics is related to the possibility of identifying imaging phenotypes associated with target expression, disease aggressiveness, treatment sensitivity or heterogeneous tumor burden.

Although diagnostic performance is good, important methodological limitations preclude clinical interpretation. Radiomics features can be substantially affected by scanner characteristics, acquisition parameters, reconstruction algorithms, segmentation procedures and preprocessing methods. Likewise, the performance of deep learning models may degrade when applied to populations or imaging protocols that differ from the training data. So, just because one dataset has high accuracy or area under the curve (AUC), it does not mean it will generalize well. External multicenter validation, standardized imaging pipelines, robust segmentation procedures, and assessment of calibration and uncertainty are thus crucial prior to considering AI-derived imaging biomarkers as reliable clinical tools [126,127].

### 8.2. AI for Patient Stratification and Therapeutic Decision-Making

The therapeutic potential of AI goes beyond the interpretation of images to the identification of patients that may be appropriate candidates for specific theranostic interventions. Machine-learning models can incorporate imaging features along with clinical, pathological, laboratory, and genomic variables to predict treatment response, progression, toxicity, or survival. Multimodal AI might be able to combine molecular imaging with genomic and pathological data to provide a more holistic view of tumor biology. These approaches may help to select patients for targeted radiopharmaceutical therapy and other molecularly guided interventions.

One major conceptual limitation, however, is the distinction between prognostic and predictive information. A model to identify patients with a poor prognosis does not necessarily identify those that would benefit from a particular treatment. Likewise, an association between an imaging feature and treatment response does not demonstrate that using that feature to guide treatment improves outcomes. Therefore, therapeutic AI needs to be supported by stronger evidence compared to traditional diagnostic prediction, such as treatment effect assessment, comparison with established clinical decision pathways, calibration and prospective assessment of whether AI-guided decisions change meaningful clinical endpoints [124].

### 8.3. AI for Response Assessment and Adaptive Therapy

AI-assisted longitudinal image analysis may help identify early response to treatment, progression or changes in tumor phenotype. Automated segmentation and quantitative assessment may potentially reduce interobserver variability and allow for consistent evaluation of serial PET/SPECT, CT, or MRI examinations. Serial imaging during radiopharmaceutical therapy may provide information about changes in tumor burden and radiopharmaceutical distribution that could support individualized treatment adaptation.

The key question is whether earlier or more accurate prediction actually makes a difference in treatment decisions. A model can predict response ahead of conventional imaging without proving that acting on that prediction improves patient outcomes. Thus, adaptive AI will require prospective evaluation, where model outputs are integrated into pre-specified clinical decision pathways and compared against current standards of care. False positive and false negative predictions also need special attention because inappropriate changes in treatment may have clinical consequences [122,123].

### 8.4. AI-Enabled Individualized Dosimetry

Personalized dosimetry represents a critical utilization of AI in theranostics, as the effectiveness and adverse effects of treatment are contingent upon the spatial and temporal allocation of radiopharmaceuticals. Artificial intelligence can help with automatic tumor and organ segmentation, quantitative image reconstruction, activity estimation, dose prediction and serial imaging data analysis. Such applications could reduce the time and computation load of patient-specific dosimetry and make possible more personalized therapeutic planning [124,125,126,127].

However, AI-based dosimetry cannot be isolated from the uncertainties of quantitative nuclear imaging. All of these, including image reconstruction, scanner calibration, acquisition timing, radiopharmaceutical kinetics, biological distribution, organ motion, and the assumptions inherent in dose modeling influence the estimation of dose. Thus, an AI-based dose estimate must be validated using well-established dosimetric methods and the associated uncertainty must be explicitly characterized. The clinical value of AI dosimetry should finally be judged not only by agreement with reference dose estimates but also by whether improved dosimetric information leads to better treatment selection, dose optimization or toxicity management [128,129].

### 8.5. Multimodal AI, Foundation Models, and Generative AI

A fundamental direction of precision theranostics is the integration of imaging, pathology, genomics and clinical records. Multimodal AI is able to discover associations between imaging phenotypes and molecular features that are undetectable by a single data source. Foundation models and generative AI also enable the processing of heterogeneous clinical information and support automated reporting, multimodal interpretation, information retrieval and clinical decision support [128].

However, the broad generalizability asserted for foundation models has not yet been demonstrated in many theranostic applications. Concerns with heterogeneous data quality, missing modalities, dataset bias, domain shift, hallucination, limited traceability, and lack of disease-specific validation remain. Furthermore, the fact that a generative model can produce a plausible clinical explanation does not mean the underlying recommendation is correct. Hence, multimodal and generative AI should be treated as decision-support tools that require validation in particular domains and appropriate supervision by humans, rather than as stand-alone therapeutic systems [123,124,125].

### 8.6. Explainable AI and Clinical Reliability

Explainable AI is particularly relevant when the output of algorithms affects diagnostic or therapeutic decisions. Feature attribution, saliency maps, attention-based visualization, confidence estimates and uncertainty analysis can assist clinicians in understanding which imaging or clinical features contribute to a prediction [123,124,125]. But we should not confuse interpretability with validity. An explanation may seem biologically plausible but be an artifact of the model or an unstable association. Hence, XAI needs to be evaluated along with reproducibility, calibration, robustness and clinical performance rather than individually to prove the reliability of the model.

Uncertainty estimation is equally important for clinical implementation. A model that can identify cases where its prediction is not reliable could be clinically more useful than a model that provides highly confident predictions for all cases. Future theranostic artificial intelligence systems should therefore include mechanisms for out-of-distribution case detection, prediction uncertainty quantification and human re-view initiation in case of insufficient model confidence [126,127,128].

### 8.7. The Translational Gap: From Algorithmic Performance to Clinical Value

The primary constraint of today’s AI-enabled theranostics is the disconnect between how well algorithms perform and the clinical value that is proven. Technical metrics (AUC, sensitivity, specificity, accuracy, dice similarity coefficient or image quality improvement) define performance for a given computational task but do not show that AI improves clinical decision-making or patient outcomes. A model can be well-discriminated but poorly calibrated, non-reproducible across institutions or clinically redundant because its information does not change treatment decisions.

Recent evidence demonstrates this limitation. An assessment of AI in oncologic theranostics in 2026 encompassed 73 full journal articles and eight conference abstracts. Among the full reports, 51 were retrospective; only 7 reported an external-type held-out evaluation, only 1 explicitly assessed calibration, and only 1 addressed uncertainty propagation, while none evaluated a prospective AI-guided treatment policy. Thus, the current evidence base is skewed toward retrospective development and workflow-support applications, rather than prospective demonstration of improved AI-guided therapeutic outcomes [128].

Hence, a clinically credible evidence pathway should include technical validation, external validation, calibration, assessment of uncertainty, human–AI comparison, prospective workflow evaluation and final demonstration of clinical utility. Importantly, the level of evidence required should be proportionate to the intended role of the AI system. Assistance in image segmentation might require demonstration of accuracy, robustness and benefit to workflow, but an AI system that determines patient eligibility, treatment selection, administered activity or treatment adaptation requires substantially stronger prospective evidence and safety assessment. Decision-curve analysis and comparison with well-established clinical models can be used to further assess whether the additional information provided by AI translates into a meaningful net clinical benefit [127,128,129].

This distinction is critical for theranostics because improvements in computational performance may not necessarily translate into improved therapeutic outcomes. Future studies should therefore report external validation, calibration, uncertainty, subgroup performance, failure modes, reproducibility, workflow effects, and patient-centered endpoints rather than relying exclusively on discrimination metrics. The transition from proof-of-concept AI to clinical theranostics will depend on demonstrating that algorithmic outputs are reproducible, clinically actionable, and capable of improving decisions without introducing unacceptable risks.

### 8.8. Digital Twins and Predictive Theranostics

Digital twins represent a new approach that integrates imaging, biological, physiological and longitudinal clinical information into patient-specific computer models to simulate disease behavior and therapeutic responses. Digital twins have been studied in oncology to optimize treatment, predict response and simulate alternative therapies [129,130,131,132]. Such models could potentially be used in theranostics to integrate tumor burden, radiopharmaceutical biodistribution, dosimetry, molecular characteristics and longitudinal response to model individualized treatment scenarios.

However, digital twins are still mostly under investigation at present. These are hampered for clinical translation by incomplete longitudinal datasets, interoperability issues, computational demands, uncertainty in biological modeling and limited validation with real-world patient outcomes. Internal or in silico validation alone cannot demonstrate that a digital twin captures an individual patient’s disease course. Prospective validation, benchmarking against existing clinical methods, and explicit uncertainty quantification will therefore be required before digital-twin systems can reliably guide therapeutic decisions [129,130,131,132].

### 8.9. Clinical and Translational Perspective

Therefore, the emerging role of AI in theranostics should be considered in a continuum from diagnostic assistance to therapeutic decision support. Radiomic and deep learning image analysis is currently closest to clinical application, while AI-guided treatment selection, adaptive therapy, foundation models and digital twins are still in earlier stages of clinical development. The primary translational hurdle across these applications is not the absence of increasingly sophisticated algorithms, but the scarcity of evidence that their outputs are reliable in varied clinical settings and deliver tangible improvements in patient care [128,129,130].

Future research should therefore focus on multicenter datasets, standardized imaging and data acquisition, external prospective validation, uncertainty-aware modeling, transparent reporting and clinically meaningful endpoints. AI should not be seen as a computational technology on its own but as part of well-established molecular imaging, dosimetric and clinical workflows. Ultimately, the long-term value of AI-enabled theranostics will depend on its ability to translate complex multimodal data into reproducible and clinically actionable information that improves diagnostic accuracy, treatment selection, individualized dosimetry, therapeutic adaptation, or patient outcomes (Table 5) [128].

### 8.10. Benefits, Limitations, and Clinical Risks of AI-Enabled Theranostics

The methods discussed in this review have complementary advantages, but also intrinsic limitations that affect their clinical translation. Sensitive and quantitative assessment of biological targets is possible with molecular imaging, but still relies on tracer kinetics, image quality, acquisition protocols, and quantitative standardization. Nanotheranostic systems can combine targeting, imaging and therapy in one platform but their clinical utility may be limited by heterogeneous tumor accumulation, biological variability, complexity of manufacture and long-term safety. While multimodal imaging can provide complementary structural, functional and molecular information, the added technical complexity may not necessarily improve clinical outcomes. These considerations suggest that the assessment of technological sophistication should be associated with reproducibility, scalability, clinical utility and patient benefit, rather than only technical capability [127].

AI presents a comparable combination of opportunity and risk. Its main advantages are: quick analysis of large and heterogeneous datasets, automated image segmentation and quantification, multimodal data integration, improved pattern recognition, individualized dosimetry, response prediction and decision support. Based on theranostics, these capabilities can enable more accurate patient selection, therapy planning, and longitudinal monitoring and adaptation. Recent findings indicate that AI is being investigated at multiple stages of the cancer theranostic workflow with varying degrees of clinical maturity across applications [127,128].

The risks of medical AI are contextual, not intrinsic to AI. Training data of poor quality or that are not representative can lead to systematic bias and lower performance in under-represented populations. Variability from scanners, acquisition protocols, patient populations, and clinical workflows can lead to dataset shift and external generalizability issues. Other concerns are the limited interpretability, overconfidence in uncertain predictions, privacy and data-security risks, automation bias, model drift, and unclear accountability when AI-assisted recommendations contribute to clinical decisions. WHO guidance published in 2025 and 2026 emphasizes that these issues require appropriate governance, ethical oversight, transparency, human accountability and continuous evaluation, not unfettered automation [133].

Most Importantly, AI should not be labeled as inherently unsafe or inherently safe for medical use at this time. Clinical risk depends on the intended use, level of autonomy, quality of the evidence, ramifications of an erroneous prediction, and the effectiveness of human supervision. The risk profile of an AI system used to assist with image segmentation differs from a system that independently recommends treatment or changes therapeutic activity. Recent evidence in cancer decision-making also suggests that seemingly high-performing artificial intelligence systems can exhibit significant variation across tasks, and could still be inappropriate for autonomous clinical decision-making without expert supervision [128].

Therefore, a risk-proportionate validation framework needs to be applied to assure the safe implementation of AI-enabled theranostics. Systems intended to influence treatment decisions should be externally and prospectively validated, calibrated, have their uncertainty assessed, be subgroup-analyzed, monitored for performance drift and compared with established clinical practice. High-consequence decisions should still involve human supervision, with clarity around who is responsible for reviewing the output of AI and how to handle disagreement between an algorithmic recommendation and clinical judgment. The right goal is not full automation but clinically governed enhancement of expert decision-making. AI may improve the consistency and depth of information available to clinicians while maintaining patient safety and accountability.

## 9. Companion Diagnostics and Precision Medicine

Companion diagnostics (CDx) is the basic building block for precision theranostics in that it combines target identification through molecular analysis with personalized treatment. In contrast with traditional diagnostics, companion diagnostics utilizes techniques such as molecular imaging, tissue biomarker tests, liquid biopsy and computational methods to establish the patient’s qualification for a certain therapy, predict the potential benefit of the therapy, select the right treatment modality, and monitor the treatment efficacy at each stage of the disease process. The use of the theranostics approach brings together diagnostics and therapy through the same molecular target [133]. The integration of molecular imaging with circulating biomarkers, artificial intelligence, and multiomics is revolutionizing the role of companion diagnostics to become dynamic support tools for decision-making that help improve treatment strategies through precision medicine.

### 9.1. Patient Stratification

Molecular patient stratification is the very first and most important step toward precision theranostics, whereby targeted treatments are delivered to those patients whose cells express high enough levels of the molecular target for the treatment. PET, SPECT, MRI, and multimodality molecular imaging make it possible to quantify the receptor density, tumor heterogeneity, disease load, and biomarker localization, which allows for better qualification for treatment and the minimization of unnecessary toxicity [134,135]. The combination of imaging biomarkers with genomics, transcriptomics, and other clinical information improves the molecular classification and selection of patients suitable for radionuclide, targeted, immunotherapy, or combined treatments.

### 9.2. Treatment Selection

Companion diagnostics have a direct bearing on the choice of therapies since they ensure the presence, accessibility, and functional significance of target molecules that can be used for personalized therapies before the administration of the therapy. Some clinically validated companion diagnostics include PSMA PET for 177Lu-PSMA therapy, somatostatin receptor PET for peptide receptor radionuclide therapy (PRRT), HER2 imaging for targeted therapies, and amyloid PET for anti-amyloid therapies in Alzheimer’s disease [42,47]. Molecular imaging in conjunction with genomic and proteomic profiling is increasingly being used for drug therapy selection based on biomarkers.

### 9.3. Response Prediction

Predicting therapeutic responses prior to the start of therapy is one of the goals of the contemporary companion diagnostics approach. Information provided by quantitative molecular imaging about receptor occupancy, tracer uptake, tumor heterogeneity, and pharmacokinetics is correlated with the effectiveness of therapy. With the help of AI-based radiomic patterns, genomic biomarkers, and ctDNA, prediction models can select responders, predict resistance pathways, estimate patients’ survival rate, and plan an optimal therapy sequence.

### 9.4. Therapy Monitoring

Theranostic imaging facilitates real-time, non-invasive assessment of the effectiveness of therapy through quantitation of changes in the expression of molecular targets, lesion metabolism, receptor binding, and tumor load during therapy [26,27]. Sequential PET/SPECT imaging, functional MRI, and radiomic analysis provide the ability to recognize response or resistance to therapy before anatomic changes become evident on conventional imaging techniques [69]. This allows for adjustments in therapy, including dosimetry of radionuclides, as well as shifting therapies as needed.

### 9.5. Adaptive Treatment

Adaptive therapy is another type of evolving precision medicine approach whereby treatment methods are dynamically adapted based on molecular imaging and circulating biomarkers. The continuous measurement of disease biology allows dynamic change in the dose of radioisotope, treatment time periods, combined treatments, and treatment schedules depending on changes in tumor properties. AI-driven dosimetry, quantitative imaging, and biomarker measurements are becoming key enablers of such highly personalized and adaptive theranostic approaches [125].

### 9.6. Biomarker-Driven Imaging

Molecular imaging with biomarkers converts molecular biomarkers into imaging biomarkers that have an application to facilitate diagnosis, treatment, and pharmacodynamics. Imaging probes with PSMA, somatostatin receptors, HER2, FAP, CXCR4, integrins, amyloid-β, tau, and inflammatory molecules offer quantifiable data about target availability and target activity and therefore serve as companion diagnostics for their respective treatments [89]. In such a fashion, the combination of molecular imaging and biomarkers makes molecular imaging an essential part of the precision medicine treatment decision-making process.

### 9.7. Liquid Biopsy Integration

Liquid biopsy has proven to be a robust method to complement companion diagnostics, since it allows for the longitudinal evaluation of the development of tumors with minimal invasiveness through ctDNA, CTCs, extracellular vesicles, cell-free RNA, and other circulating biomarkers. Liquid biopsy provides advantages that are not available in traditional tissue biopsy because liquid biopsy is able to capture molecular heterogeneity from multiple sites of the body and track changes related to resistance mutations, treatment response, and recurrence detection [95,96]. Integration of liquid biopsy with molecular imaging provides complementary spatial and molecular information, substantially strengthening patient stratification and therapeutic monitoring within precision oncology.

### 9.8. Minimal Residual Disease (MRD)

MRD has emerged as one of the most promising applications of companion diagnostics as well as an indication of the residual tumor burden that is still present despite curative treatment, but which cannot be detected with the help of traditional imaging methods. Using ctDNA tests, methylation analysis, and next-generation sequencing techniques, it can be possible to detect the presence of residual disease months before its visualization. MRD evaluation in the future will be achieved through the incorporation of molecular imaging, liquid biopsy, and AI-powered predictive modeling, which will result in efficient identification of molecular relapse, determination of the optimal treatment time, and personalized surveillance for precision cancer management [119].

## 10. Smart, Programmable, and Emerging Theranostic Systems

Advanced and stimulus-responsive theranostic systems denote a recognized technological pathway within precision medicine, rather than an advancement that surfaced following artificial intelligence. These platforms were developed to overcome limitations of traditional diagnose-and-treat approaches, by responding to disease-associated biological or externally applied stimuli and integrating sensing, imaging, drug delivery or therapeutic activation in a single system. Recent advances include activatable probes, self-reporting nanocarriers, programmable nucleic acid systems, engineered cells, synthetic biological circuits and living medicines. Current research is progressively extending these established concepts via programmable biology and AI-assisted design, optimization and multimodal integration.

The conceptual difference is thus between the theranostic platform and the computational or biological intelligence applied to the theranostic platform. Stimulus-responsive systems offer ways of sensing and reacting to biological conditions, and artificial intelligence can help in their design, behavior prediction, quantitative interpretation, patient selection and adaptive decision-making. This convergence is exemplified by recent AI-driven nanotheranostic research, where machine learning has been incorporated into nanoparticle design, biodistribution modeling, treatment optimization, uncertainty assessment, and personalized therapeutic approaches [123,124,125].

### 10.1. Activatable Probes and Self-Reporting Nanoparticles

Activatable theranostic systems are engineered to be relatively inert until they are exposed to disease-associated conditions, including altered pH, reactive oxygen species, hypoxia, specific enzymes or externally applied physical stimuli. Activation can simultaneously produce an imaging signal and induce therapeutic release, thus improving disease selectivity and minimizing background activity. Self-reporting nanoparticles take this idea one step further by integrating therapeutic delivery with signals that are capable of reporting localization, cargo release, target engagement or treatment-associated changes. The main advantage is therefore the integration of biological sensing and functional monitoring on a single platform. However, a signal from an imaging probe does not necessarily mean efficient drug delivery or therapeutic response, and the differences between probe activation, biological activity and clinical outcome are still significant translational limitations [95].

### 10.2. Responsive Nanomedicine

In responsive nanomedicine, nanocarriers that can release their payload in response to specific external or internal triggers such as pH, redox conditions, hypoxia, enzyme activity, ATP, and inflammation are used for effective and selective delivery of drugs. The use of stimulus-responsive nanocarriers enables an increase in drug efficacy and reduction in toxic effects via regulation of drug release location and time [70]. Modern trends in development of nanotechnologies have resulted in the design of multifunctional nanosystems that possess multiple functions such as drug release, imaging, immune modulation, gene delivery, and biosensing [104].

### 10.3. CRISPR Imaging and RNA Imaging

Imaging using the CRISPR system has been demonstrated to be an innovative tool in visualizing the genomic structure, gene regulation, and molecular interactions within live cells without affecting the genomic content. Catalytically deficient Cas proteins along with fluorescence or radionucleotide labels have facilitated precise imaging of targeted DNA and RNA, which is useful in real-time study of molecular mechanisms in the context of diseases [78]. Advances in RNA imaging, such as molecular beacons, RNA aptamers, fluorescent RNA probes, and CRISPR-based RNA nanoplatforms, have made it possible to visualize and deliver RNA therapeutics. Together, these technologies establish an integrated platform for precision diagnostics, genome-guided therapy, and programmable molecular intervention [79].

### 10.4. Living and Cell-Based Theranostics

Living and cell-based theranostics is a more general class of systems, where viable biological entities are used as therapeutic agents, delivery vehicles, sensors, or a combination of these. Mammalian cells, immune cells, stem or stromal cells, macrophages, engineered bacteria and other living systems can use intrinsic homing mechanisms or engineered functions to deliver therapeutics to disease sites that are difficult to reach with conventional carriers. Imaging reporters may also be integrated to monitor cellular localization, persistence, viability, or therapeutic activity. Recent work has highlighted the potential of engineered cellular systems for targeted delivery and therapeutic intervention while also emphasizing the challenges associated with heterogeneous biological behavior, immune interactions and control of in vivo function [90,91].

Living medicines are an emerging class within this broader category, including engineered mammalian cells, bacteria, fungi and viruses. The key attribute of the therapeutic entity is that it can sense, respond to, or alter its biological environment. This differentiates living medicines from passive nanocarriers and traditional biologics, but also puts extra demands on maintaining viability, controlling replication and metabolism, regulating biodistribution, and restricting immunogenicity. However, these biological properties present unique delivery and safety challenges that must be addressed before broad clinical implementation, as has been pointed out in recent analyses.

A particularly important example is that of engineered microorganisms. Bacteria can exploit tumor-associated conditions and have been engineered to deliver therapeutic payloads, modulate the tumor microenvironment and activate treatment in the presence of disease-associated signals. Synthetic-biology approaches offer better targeting and controllability. However, unpredictable behavior, safety, microbiome interactions and heterogeneous tumor environments are major translational barriers [90,91,120].

### 10.5. Immune Theranostics and Synthetic Biology

The field of immune theranostics combines molecular imaging with immunotherapy to understand immune cell behavior, response to treatment, and personalization of immunotherapy. Immune checkpoint markers, markers of activated T lymphocytes, macrophage markers, and stromal markers are some of the imaging markers used to quantitatively evaluate immune activation and efficacy of therapy. On the other hand, developments in the field of synthetic biology are leading to programmable therapeutic systems using genetically modified cells, synthetic gene circuits, molecular sensors, and logic-gated therapies, which will work only under specific disease microenvironment.

### 10.6. Protein Engineering and DNA Nanotechnology

The advent of protein engineering technology has resulted in the creation of very stable antibodies, nanobodies, affibodies, enzyme proteins, cytokines, and ligands, which have enhanced specificity, pharmacokinetics, and therapeutic effects. Moreover, DNA nanotechnology allows for the creation of nanostructures that are programmable by virtue of DNA origami and nucleic acid self-assembly and thus provide well-organized carriers that can perform multiplex molecular recognition, drug delivery, sensing, and multimodal imaging [119]. The combination of engineered proteins with DNA nanostructures will enable the creation of highly programmable theranostic systems.

### 10.7. Critical Perspective on Smart Theranostics

The major conceptual advance of smart theranostic systems is the transition from passive delivery to biologically responsive and potentially adaptive therapeutic systems. Nonetheless, enhanced programmability does not inherently equate to augmented clinical worth. Any additional sensing, targeting, imaging or therapeutic function can introduce new sources of biological variability, manufacturing complexity and regulatory uncertainty. This limitation is especially important for living and programmable systems, where a purely physicochemical description is not always enough to predict biological behavior. The recent literature on living medicines and engineered microorganisms points to the necessity of control over viability, replication, biodistribution, immunogenicity, containment, and therapeutic activity. Future development should, therefore, focus on validated relationships between the biological signal being detected, the therapeutic response elicited and the clinically relevant outcome. Ultimately, the translational value of smart theranostics will not be measured by the number of functions integrated in a platform, but by the ability of those functions to behave predictably in vivo, to be manufactured reproducibly and to offer a measurable benefit over established therapeutic approaches.

## 11. Current Challenges and Limitations of Theranostic Platforms

Despite the great advances in the fields of molecular imaging, targeted therapy, nanotechnology, radiopharmaceuticals, and computational approaches, the translation of theranostic platforms into routine clinical practice is limited by inter-related biological, technological, regulatory and clinical constraints. The main difficulty is that performance observed in controlled laboratory settings does not necessarily predict behavior in heterogeneous patient groups. The development of theranostics, therefore, calls increasingly for the simultaneous consideration of biological validity, quantitative reliability, manufacturability, safety, and clinical utility.

### 11.1. Biological Heterogeneity and Target Variability

The heterogeneity of tumor disease is still a major limitation for precision theranostics. Molecular targets can vary between patients, between primary and metastatic lesions and during treatment course. Spatial heterogeneity can lead to differential tracer uptake, non-uniform therapeutic delivery, and discordance between imaging-defined target expression and actual therapeutic susceptibility. These limitations are especially relevant for targeted radiopharmaceutical therapy and nanotheranostics, where target density, vascular access, tumor penetration and cellular internalization can all significantly affect therapeutic exposure. Thus, a positive molecular imaging signal should not be automatically considered as a uniform therapeutic sensitivity [135,136].

### 11.2. Quantitative Imaging and Dosimetry

Patient selection, biodistribution assessment, response evaluation and individualized dosimetry are all dependent on quantitative imaging. The reliability of quantitative imaging is determined by the acquisition protocols, reconstruction methods, scanner calibration, attenuation and scatter correction, image timing, segmentation and kinetic assumptions. In radiopharmaceutical therapy, administered activity is not a good descriptor of tumor or normal-tissue exposure. Therefore, patient-specific dosimetry necessitates accurate estimation of activity distribution and absorbed dose, but the application could be complicated by uncertainty in biological kinetics and repeated imaging. Standardized quantitative imaging and efficient dosimetry have been identified as important priorities for advancing precision radiopharmaceutical therapy in recent reviews.

### 11.3. Platform Complexity, Manufacturing, and Reproducibility

The appeal of theranostic platforms is their multifunctionality; however, this may also increase the complexity of formulation and introduce additional critical quality attributes. Nanoparticle size, surface characteristics, drug or radionuclide loading, stability, release behavior, targeting ligands and imaging components can all affect biological performance. However, it can be difficult, especially for complex biological, cellular or living systems, to retain these properties during scale-up and storage. Thus, adding more functional components does not necessarily improve clinical performance but may increase manufacturing variability and regulatory burden [137].

### 11.4. Safety and Off-Target Effects

Theranostic interventions can produce risks from both the diagnostic and the therapeutic side. Nanomaterials may show unexpected tissue accumulation, immune interactions or long-term retention. For radionuclide-based approaches, radiation exposure to normal organs must be considered. Cumulative toxicity may result from repeated administration when target expression is present in normal tissues and off-target therapeutic uptake may occur. Living systems and cell-based systems also present additional problems of persistence, immune response, uncontrolled biological activity and containment for some engineered systems. Hence, safety assessment needs to extend beyond short-term efficacy to include biodistribution, long-term toxicity, immune effects and clinically relevant exposure.

### 11.5. Standardization and Clinical Validation

Heterogeneity in image protocols, radiotracer preparation, nanoparticle characterization, methodology of dosimetry and outcome definitions hampers the comparison between studies and adds to the uncertainty on reproducibility. Many of the novel theranostic platforms are still mostly supported by preclinical studies or early clinical studies and it is difficult to ascertain if any improvements in imaging or pharmacokinetic parameters translate into meaningful patient benefit. Standardized protocols, multicenter validation, predefined endpoints and prospective clinical studies are therefore needed to establish the reproducibility and clinical utility of promising platforms [119].

### 11.6. Infrastructure, Accessibility, and Health-System Constraints

The implementation of clinical theranostics demands a cohesive infrastructure, potentially comprising molecular imaging facilities, radiopharmacy functions, radionuclide provision, dosimetry knowledge, expert personnel, radiation safety measures, and collaborative clinical teams. Even when a therapy is shown to be clinically effective, limited availability of the radionuclide, production capacity, specialized equipment and geographical access can limit implementation. Such constraints are of particular importance in the case of complex personalized approaches requiring repeated imaging and individual dosimetry. Thus, platform development should consider the healthcare-system feasibility along with biological and technological performance.

### 11.7. Evidence-to-Clinical-Value Gap

A persistent constraint in theranostic research is the differentiation between proving the technical efficacy of a platform and establishing its enhancement of clinical decision-making or patient outcomes. Technological advances such as enhanced imaging sensitivity, better targeting, higher drug loading, smaller particle size, or improved computational performance do not necessarily translate into measurable clinical benefit. Therefore, the most important translational question is whether the integrated platform enhances established clinical pathways in terms of treatment effectiveness, toxicity, patient selection, quality of life, or other clinically meaningful outcomes.

Generally, the main limitations of theranostics are interconnected rather than isolated. Biological heterogeneity can limit targeting efficiency, imperfect quantitative imaging can limit dosimetry, platform complexity can limit manufacturing and reproducibility, and infrastructure and regulatory limitations can limit clinical accessibility (Table 6). Solutions to these challenges must be strategically developed and span the entire continuum from biological target identification and platform design to quantitative assessment, manufacturing, clinical validation and patient outcomes. This integrated perspective forms the basis for realistic priorities of the future development of precision theranostics [136].

## 12. Future Directions

In the future, theranostic systems will make extensive use of personalized nanomedicine, which will have the capability of patient-specific loading, controlled release, and multimodal imaging to optimize their efficacy and reduce systemic toxicity. The use of digital twins with longitudinal molecular imaging, genomics, pathology, and clinical information will make it possible to create a virtual model of the patient for treatment simulation, dose planning, and prediction of therapeutic success. In parallel, AI-assisted therapy will be used to automate patient stratification, treatment response prediction, and adaptive dosing.

Future imaging systems based on self-reliant systems that use artificial intelligence as well as robotics have been suggested to assist in the process of obtaining images in real time with less dependence on operators. Quantum imaging is anticipated to enhance the sensitivity, spatial resolution, and low-dose detection of molecules. Single-cell theranostics will revolutionize cellular characterization and personalized medicine for the treatment of cellular heterogeneity. The integration of spatial multiomics with molecular imaging will ensure comprehensive mapping of genomics, transcriptomics, proteomics, and metabolomics of whole tissues [96].

Real-time adaptive therapy, in which treatment is adaptively adjusted in accordance with the evolution of the disease through imaging, will be another aspect that the next generation of theranostics will focus on. Wearable and implanted imaging sensors can facilitate the longitudinal and remote assessment of the efficacy of treatment, while image-guided therapy through nanocarriers, focused ultrasonics, magnetic targeting, and robotics can enhance therapeutic precision. Overall, these innovations are anticipated to establish the closed-loop precision medicine concept [37].

**Figure 4:**
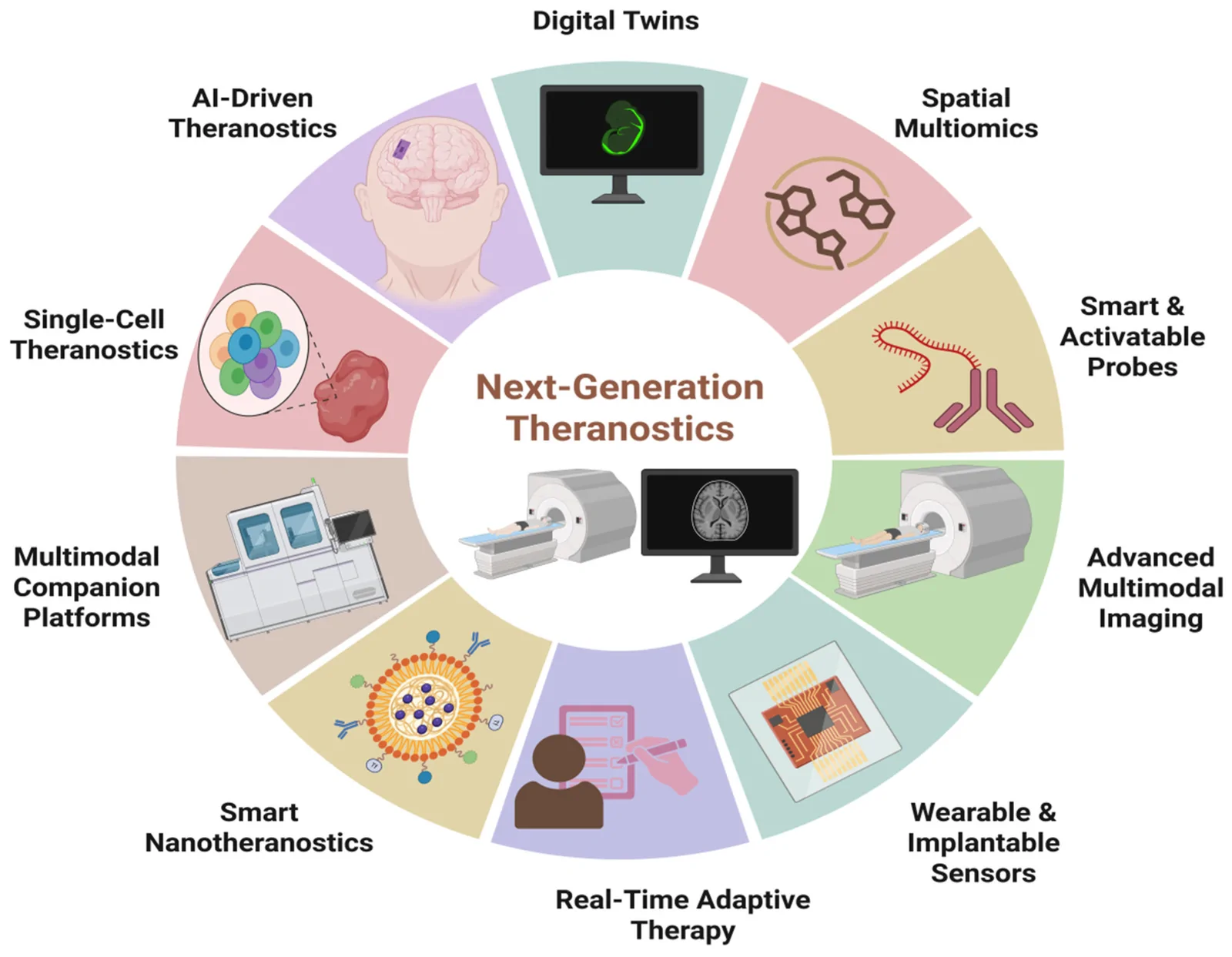
Next-generation theranostics: future perspectives. Created in BioRender. Rabbani, S.A. (2026) https://BioRender.com/5d5s1k2.

Even with considerable advances, some of the key obstacles have continued to restrict clinical applications. Biological diversity and fluctuating target expressions make the imaging less accurate and the treatment less effective, whereas off-target distribution and inadvertent exposure to healthy tissues play major roles in toxicity. The key aspect in radiotheranostics is the fine-tuning of the radiation dose based on individual dosimetry. On the other hand, regulatory harmonization is needed to facilitate approval procedures for diagnostic–therapy combination devices, especially involving such innovations as artificial intelligence, nanotechnology, gene editing, and living therapies. Manufacturing difficulties including GMP manufacture, the availability of radioisotopes, quality control, and logistics continue to be obstacles for wider application, as depicted in Figure 4.

## 13. Conclusions

The emergence of theranostic imaging has completely revolutionized the concept of precision medicine, as it combines molecular diagnosis, targeted therapy, and treatment assessment into one clinical approach. The clinical success of radiotheranostics, especially the use of PSMA- and somatostatin receptor-targeted treatments, has proven that molecular imaging is useful not only for diagnosis but also for predicting and treating conditions, in addition to being a prognostic and therapeutic tool. At the same time, nanotheranostics, multimodal imaging, artificial intelligence, companion diagnostics, and biomarker-based patient stratification have widened the application of theranostics into many areas other than oncology.

The coming era of innovations in theranostics will emerge out of the synergies of artificial intelligence-based clinical decision-making systems, digital twins, spatial multiomics, single-cell biology, programmable nanomedicine, genome editing, synthetic biology, and intelligent image-guided drug delivery. The result of these innovations would be the transformation of theranostics from its current imaging-based mode into an adaptive healthcare system that is continuously evolving and able to predict the course of the disease and modify therapy based on real-time molecular responses.

As a whole, theranostic imaging will shift from being a niche strategy within the realm of nuclear medicine into a precision medicine approach that harmoniously incorporates molecular diagnostics, therapies, intelligent imaging, and intelligent clinical management. Further developments in the fields of molecular targeting, intelligent imaging, radiopharmacy, nanotechnology, and artificial intelligence are predicted to result in theranostics becoming one of the cornerstones of advanced personalized medicine.

## Figures and Tables

**Table 1:** Comparison of molecular imaging modalities used in theranostics.

Imaging Modality	Imaging Principle	Imaging Agent/Contrast	Sensitivity	Spatial Resolution	Tissue Penetration	Quantification	Major Clinical Applications	Role in Theranostics	Major Advantages	Major Limitations	Clinical Translation	Refs.
PET	Detection of positron annihilation photons	^18^F, ^68^Ga, ^64^Cu, ^89^Zr radiotracers	Very High (10-11–10-12 M)	3–5 mm	Deep-tissue imaging; whole-body coverage; attenuation and scatter correction required	Excellent	Oncology, neurology, cardiology, infection	Patient selection, dosimetry, radionuclide therapy guidance, response monitoring	Highest molecular sensitivity, quantitative imaging, whole-body assessment	Ionizing radiation, high cost, cyclotron/radiochemistry requirements	Routine clinical practice	[40]
SPECT	Gamma photon detection	^99^mTc, ^111^In, ^123^I, ^177^Lu	High	7–12 mm	Deep-tissue imaging; whole-body coverage; attenuation and scatter affect quantitative accuracy	Moderate	Oncology, cardiology, thyroid disorders	Post-therapy imaging, personalized dosimetry	Widely available, inexpensive radionuclides, therapeutic isotope imaging	Lower sensitivity and resolution than PET	Routine clinical practice	[41]
MRI	Nuclear magnetic resonance of hydrogen nuclei	Gadolinium, iron oxide, manganese nanoparticles	Moderate	Excellent (0.1–1 mm)	Deep-tissue imaging; field-of-view- and hardware-dependent	Moderate	Brain, soft tissues, musculoskeletal, cardiovascular	Nanotheranostics, drug delivery, image-guided therapy	Superior soft-tissue contrast, no ionizing radiation, multiparametric imaging	Lower molecular sensitivity, longer acquisition time	Routine clinical practice	[42]
Optical Imaging (Fluorescence/NIR)	Fluorescence emission from targeted probes	Organic fluorophores, NIR-I/NIR-II dyes, quantum dots	Very High	10–100 μm	Limited tissue penetration; primarily superficial or intraoperative applications	Limited	Image-guided surgery, preclinical imaging, endoscopy	Surgical guidance, activatable probes, intraoperative theranostics	Real-time imaging, inexpensive, high sensitivity	Limited penetration depth, tissue autofluorescence	Clinical and preclinical	[43]
Ultrasound Molecular Imaging	Reflection of high-frequency sound waves with targeted microbubbles	Targeted microbubbles, nanobubbles	Moderate	100–500 μm	Limited depth; strongly dependent on tissue composition and acoustic attenuation	Limited	Cardiovascular disease, liver disease, vascular imaging	Drug delivery, sonoporation, BBB opening	Portable, inexpensive, real-time, radiation-free	Operator dependence, limited molecular sensitivity	Clinical (selected applications)	[44]
Photoacoustic Imaging (PAI)	Laser-induced ultrasound generation	Gold nanoparticles, organic dyes, semiconducting polymers	High	50–300 μm	Intermediate depth; dependent on wavelength and tissue optical/acoustic properties	Moderate	Oncology, vascular imaging, inflammation	Photothermal therapy guidance, smart nanotheranostics	Combines optical contrast with ultrasound penetration	Limited whole-body imaging, technology still evolving	Early clinical translation	[45]
Raman Imaging (SERS)	Inelastic light scattering	SERS nanoparticles, Raman reporters	Extremely High (single-molecule possible)	<1 μm	Low (few mm)	Moderate	Surgical oncology, pathology, biosensing	Multiplex molecular imaging, image-guided surgery	Exceptional molecular specificity, multiplexing capability	Weak intrinsic signal, shallow penetration	Mostly preclinical/early clinical	[46]
Multimodal Imaging (PET/CT, PET/MRI, SPECT/CT, PET/Optical, MRI/PAI)	Integration of complementary imaging modalities	Dual- or multimodal probes	Very High	Excellent	Whole body	Excellent	Precision oncology, neurology, cardiovascular imaging	Companion diagnostics, image-guided therapy, adaptive treatment	Combines anatomical, molecular and functional information	Probe design complexity, higher cost, regulatory challenges	Rapidly expanding clinical use	[47]

**Table 2:** Design principles and components of theranostic imaging agents.

Design Component	Design Strategy	Representative Examples	Primary Function	Advantages	Major Challenges	Translational Status	Refs.
Targeting ligand	Monoclonal antibodies	Trastuzumab, Cetuximab	High-specificity receptor targeting	Excellent specificity	Large size, slow clearance	Clinical	[56]
Targeting ligand	Antibody fragments	Fab, scFv, Nanobody	Improved tissue penetration	Faster imaging	Lower circulation time	Clinical/Early clinical	[57]
Targeting ligand	Peptides	PSMA-617, DOTATATE, RGD	Receptor-mediated targeting	Rapid penetration	Enzymatic degradation	Clinical	[56]
Targeting ligand	Small molecules	Fibroblast Activation Protein Inhibitor (FAPI), PSMA inhibitors	Target-specific imaging	Simple synthesis	Limited payload capacity	Clinical	
Targeting ligand	Aptamers	AS1411	Nucleic acid-based recognition	Low immunogenicity	Nuclease degradation	Preclinical	[58]
Targeting ligand	Affibodies	HER2 affibody	High-affinity protein targeting	Small size	Short half-life	Early clinical	[57]
Imaging moiety	PET radionuclides	^18^F, ^68^Ga, ^64^Cu, ^89^Zr	Quantitative molecular imaging	Highest sensitivity	Radiation exposure	Clinical	[56]
Imaging moiety	SPECT radionuclides	^99^mTc, ^111^In	Functional imaging	Widely available	Lower sensitivity	Clinical	
Imaging moiety	MRI contrast agents	Gd, SPIONs, Mn	Anatomical and functional imaging	Excellent soft-tissue contrast	Low molecular sensitivity	Clinical	[59]
Imaging moiety	Optical fluorophores	ICG, Cy5.5, IRDye800	Fluorescence imaging	Real-time imaging	Limited penetration	Clinical/Preclinical	
Imaging moiety	Photoacoustic probes	Gold nanorods, semiconducting polymers	Deep optical imaging	High contrast	Emerging technology	Early clinical	
Imaging moiety	Raman probes	SERS nanoparticles	Molecular fingerprinting	Multiplex detection	Specialized instrumentation	Preclinical	
Therapeutic payload	β-emitting radionuclides	^177^Lu, ^90^Y	Targeted radiotherapy	Proven efficacy	Organ dosimetry	Clinical	[56]
Therapeutic payload	α-emitting radionuclides	^225^Ac, ^212^Pb	High-LET radiotherapy	Highly potent	Production limitations	Clinical/Early clinical	
Therapeutic payload	Chemotherapeutics	Doxorubicin, Paclitaxel	Cytotoxic therapy	Established efficacy	Systemic toxicity	Clinical	
Therapeutic payload	Gene therapeutics	siRNA, mRNA, CRISPR	Gene modulation	Precision therapy	Delivery barriers	Early clinical	
Therapeutic payload	Immunotherapeutics	Cytokines, checkpoint inhibitors	Immune activation	Durable responses	Immune toxicity	Clinical	
Therapeutic payload	Phototherapy agents	Photosensitizers, photothermal agents	PDT/PTT	Spatial control	Light penetration	Clinical/Preclinical	[60]
Stimulus-responsive design	pH-responsive	Charge-switch nanoparticles	Tumor-selective activation	Reduced off-target toxicity	Variable tumor pH	Preclinical	[61]
Stimulus-responsive design	Enzyme-responsive	MMP-cleavable systems	Disease-specific release	High specificity	Enzyme variability	Preclinical	[59]
Stimulus-responsive design	ROS-responsive	Thioketal nanoparticles	Oxidative stress-triggered release	Selective activation	Biological variability	Preclinical	[60]
Stimulus-responsive design	Hypoxia-responsive	Nitroimidazole systems	Hypoxic tumor targeting	Precision therapy	Heterogeneous hypoxia	Preclinical	[62]
Stimulus-responsive design	External-triggered	Ultrasound, magnetic field, NIR light	Controlled drug release	Temporal precision	Equipment dependency	Early clinical	[59]
Pharmacokinetic optimization	PEGylation	PEG-coated nanoparticles	Prolong circulation	Reduced immune clearance	Accelerated blood clearance phenomenon	Clinical	[63]
Pharmacokinetic optimization	Albumin binding	Evans Blue derivatives	Enhanced tumor accumulation	Increased half-life	Variable albumin binding	Clinical/Early clinical	[64]
Pharmacokinetic optimization	Biomimetic coating	Cell membrane-coated nanoparticles	Immune evasion	Improved biocompatibility	Manufacturing complexity	Preclinical	[62]
Pharmacokinetic optimization	Surface charge engineering	Zwitterionic/charge-switch systems	Biodistribution optimization	Better cellular uptake	Stability control	Preclinical	[61]
Pharmacokinetic optimization	Cleavable linkers	Acid-, enzyme-, redox-cleavable	Controlled intracellular release	Reduced systemic toxicity	Linker stability	Clinical/Early clinical	[65]
Multifunctional integration	Multimodal theranostic construct	Targeting ligand + imaging probe + therapeutic payload + responsive linker	Simultaneous diagnosis, therapy and monitoring	Precision medicine, real-time feedback	Complex manufacturing, regulatory approval	Translational/Clinical	[63]

**Table 3:** Clinically approved and investigational theranostic imaging agents.

**(A) Clinically Approved Theranostic Imaging Agents**								
**Molecular Target**	**Diagnostic Imaging Agent**	**Therapeutic Agent**	**Imaging Modality**	**Primary Disease**	**Regulatory Status**	**Clinical Significance**		**Refs.**
PSMA	^68^Ga-PSMA-11	^177^Lu-PSMA-617 (Pluvicto^®^)	PET/CT	Metastatic castration-resistant prostate cancer (mCRPC)	FDA (2022; expanded 2025), EMA	Landmark precision radiotheranostic; standard-of-care for PSMA-positive mCRPC		[30,80]
PSMA	^18^F-DCFPyL (Pylarify^®^, Lantheus Medical Imaging, Billerica, MA, USA)	^177^Lu-PSMA-617	PET/CT	mCRPC	FDA-approved companion imaging	High-resolution patient selection before PSMA radioligand therapy		
PSMA	^18^F-rhPSMA-7.3 (POSLUMA^®^, Blue Earth Diagnostics Ltd., Oxford, UK)	^177^Lu-PSMA-617	PET/CT	Prostate cancer recurrence	FDA-approved	Alternative PSMA imaging companion diagnostic		
Somatostatin receptor (SSTR2)	^68^Ga-DOTATATE (Netspot^®^, Novartis Pharma AG, Basel, Switzerland)	^177^Lu-DOTATATE (Lutathera^®^)	PET/CT	Gastroenteropancreatic neuroendocrine tumors (GEP-NETs)	FDA (2018), EMA	First modern peptide receptor radionuclide therapy (PRRT); practice-changing		[80,81]
Somatostatin receptor (SSTR2)	^68^Ga-DOTATOC	^177^Lu-DOTATATE	PET/CT	NETs	EMA/International clinical use	Widely used European PRRT imaging pair		[82]
Somatostatin receptor (SSTR2)	^64^Cu-DOTATATE	^177^Lu-DOTATATE	PET/CT	Neuroendocrine tumors	FDA-approved imaging	Longer half-life improves logistics and lesion detection		
Norepinephrine transporter (NET)	^123^I-MIBG	^131^I-Iobenguane (Azedra^®^, Lantheus Medical Imaging, Billerica, MA, USA)	SPECT/CT	Pheochromocytoma and Paraganglioma	FDA (2018)	First targeted radiotherapy for metastatic PPGL		[83]
Sodium-Iodide Symporter (NIS)	^123^I Sodium Iodide	^131^I Sodium Iodide	SPECT	Differentiated thyroid carcinoma	Worldwide standard	Oldest and most successful theranostic paradigm		[84]
Bone hydroxyapatite (osteoblastic metastases)	^99^mTc-MDP	^223^Ra-Dichloride (Xofigo^®^, Bayer AG, Leverkusen, Germany.)	Bone scintigraphy/SPECT	Bone metastatic prostate cancer	FDA (2013), EMA	First approved alpha-emitter improving overall survival		[81]
Bone hydroxyapatite	^99^mTc-MDP	^89^Sr-Chloride (Metastron^®^, GE Healthcare Ltd., Amersham, UK)	Bone scintigraphy	Painful bone metastases	Approved (historical clinical use)	Beta-emitter for palliation of skeletal metastases		
Bone hydroxyapatite	^99^mTc-MDP	^153^Sm-Lexidronam (Quadramet^®^, Lantheus Medical Imaging, Billerica, MA, USA)	Bone scintigraphy	Bone metastases	Approved (historical)	Targeted palliation for osteoblastic skeletal metastases		
CD20	^111^In-Ibritumomab Tiuxetan	^90^Y-Ibritumomab Tiuxetan (Zevalin^®^, Acrotech Biopharma LLC, East Windsor, NJ, USA)	SPECT	B-cell Non-Hodgkin lymphoma	FDA (2002)	First radioimmunotherapy demonstrating targeted antibody theranostics		[85]
CD20	^131^I-Tositumomab	^131^I-Tositumomab (Bexxar^®^, GlaxoSmithKline LLC, Research Triangle Park, NC, USA)	Gamma imaging	Non-Hodgkin lymphoma	Historical FDA approval (withdrawn commercially)	Pioneering radioimmunotherapy establishing antibody-based theranostics		
Liver arterial vasculature	^99^mTc-MAA simulation	^90^Y Glass/Resin Microspheres (TheraSphere^®^, Boston Scientific Corporation, Marlborough, MA, USA, SIR-Spheres^®^, Sirtex Medical Pty Ltd., Sydney, Australia)	SPECT/CT	Hepatocellular carcinoma and liver metastases	FDA/HDE and international approvals	Personalized radioembolization guided by pre-treatment perfusion imaging		[86]
Bone metastasis targeting	Bone scintigraphy (^99^mTc agents)	^223^Ra-Dichloride (Xofigo^®^)	SPECT	Castration-resistant prostate cancer with symptomatic bone metastases	FDA/EMA	Precision alpha-particle therapy with imaging-guided patient selection		[81]
(B) Late-Stage Clinical (Phase II/III) Theranostic Agents								
**Molecular Target**	**Diagnostic Imaging Agent**	**Therapeutic Agent**	**Imaging Modality**	**Primary Indication**	**Clinical Stage**	**Major Advantages**	**Current Limitations**	**Refs.**
Fibroblast Activation Protein (FAP)	^68^Ga-FAPI-46	^177^Lu-FAPI-46	PET/CT	Multiple solid tumors	Phase II	Pan-tumor stromal targeting; excellent tumor-to-background ratio	Limited tumor retention	[77,87]
Fibroblast Activation Protein (FAP)	^68^Ga-FAP-2286	^177^Lu-FAP-2286	PET/CT	Pancreatic, breast, colorectal and other solid tumors	Phase II	Improved tumor retention and dosimetry	Long-term efficacy under evaluation	
Fibroblast Activation Protein (FAP)	^68^Ga-EB-FAPI	^177^Lu-EB-FAPI	PET/CT	Advanced epithelial cancers	Phase II	Albumin binding prolongs circulation	Higher marrow exposure	[88]
CXCR4	^68^Ga-Pentixafor	^177^Lu-Pentixather	PET/CT	Multiple myeloma, lymphoma	Phase II	Excellent hematologic tumor targeting	Hematologic toxicity requires monitoring	[78,89]
CXCR4	^68^Ga-Pentixafor	^90^Y-Pentixather	PET/CT	Hematologic malignancies	Phase II	High radiation dose delivery before stem-cell transplantation	Myeloablative toxicity	
PSMA	^18^F-rhPSMA-7.3	^225^Ac-PSMA-617	PET/CT	Metastatic prostate cancer	Phase II/III	High efficacy in PSMA-positive disease resistant to β-therapy	Xerostomia, radionuclide availability	[56]
PSMA	^68^Ga-PSMA-11	^212^Pb-PSMA	PET/CT	Advanced prostate cancer	Phase II	Short-range α-particle therapy with reduced off-target toxicity	Production challenges	
Somatostatin Receptor (SSTR2)	^68^Ga-DOTA-JR11	^177^Lu-DOTA-JR11	PET/CT	Neuroendocrine tumors	Phase III	Receptor antagonist improves tumor uptake	Kidney dosimetry optimization required	[90]
Somatostatin Receptor (SSTR2)	^68^Ga-OPS202	^177^Lu-OPS201	PET/CT	Gastroenteropancreatic NETs	Phase III	Higher lesion detection than agonists	Limited long-term outcome data	
Carbonic Anhydrase IX (CAIX)	^89^Zr-Girentuximab	^177^Lu-Girentuximab	PET/CT	Clear-cell renal cell carcinoma	Phase III	Highly specific ccRCC targeting	Slow antibody pharmacokinetics	
HER2	^89^Zr-Trastuzumab	^177^Lu-Trastuzumab	PET/CT	HER2-positive breast and gastric cancer	Phase II	Enables HER2 expression mapping and targeted radionuclide therapy	Hematologic toxicity	[56]
Gastrin-Releasing Peptide Receptor (GRPR)	^68^Ga-RM2	^177^Lu-RM2	PET/CT	Prostate and breast cancer	Phase II	High sensitivity for GRPR-positive tumors	Variable receptor expression	[91]
Estrogen Receptor (ER)	^18^F-FES	^177^Lu-Estrogen receptor-targeted ligand	PET/CT	ER-positive metastatic breast cancer	Phase II	Functional receptor imaging for personalized endocrine therapy	Early therapeutic validation	[92]
DLL3	^89^Zr-labeled DLL3 antibody	^177^Lu-DLL3 antibody	PET/CT	Small-cell lung cancer	Phase II	Highly selective neuroendocrine tumor targeting	Early efficacy data	[91]
Fibroblast Activation Protein (FAP)-RGD dual target	^68^Ga-FAPI-RGD	^177^Lu-FAPI-RGD	PET/CT	Advanced solid tumors	Phase II	Simultaneous stromal and angiogenic targeting	Clinical validation ongoing	[93]
(C) Early Clinical (Phase I/II) Theranostic Imaging Agents								
**Molecular Target**	**Diagnostic Imaging Agent**	**Therapeutic Agent**	**Imaging Modality**	**Major Indication(s)**	**Clinical Stage**	**Major Advantages**		**Refs.**
FAP	^68^Ga-FAPI-04	^177^Lu-FAPI-04	PET/CT	Advanced solid tumors	Phase I	First-generation FAP theranostic		[87]
FAP	^68^Ga-FAPI-46	^90^Y-FAPI-46	PET/CT	Pan-cancer	Phase I	Higher therapeutic radiation dose		
FAP	^68^Ga-(SA.FAPi)_2_	^177^Lu-(SA.FAPi)_2_	PET/CT	Advanced epithelial cancers	Phase I	Dimerization improves tumor retention		[93]
FAP	^68^Ga-3BP-3940	^177^Lu-3BP-3940	PET/CT	Refractory solid tumors	Phase I	Improved pharmacokinetics		
FAP	^68^Ga-FAPI-RGD	^177^Lu-FAPI-RGD	PET/CT	Pan-cancer	Phase I	Dual targeting of FAP and integrin αvβ3		[94]
GRPR	^68^Ga-NeoBOMB1	^177^Lu-NeoBOMB1	PET/CT	Prostate, breast cancer	Phase I/II	High GRPR affinity with favorable imaging		[95]
GRPR	^68^Ga-RM2	^177^Lu-RM2	PET/CT	Prostate cancer	Phase I/II	Excellent lesion detection		
Integrin αvβ3	^68^Ga-RGD	^177^Lu-RGD	PET/CT	Glioma, breast cancer	Phase I	Angiogenesis-targeted theranostics		
HER2	^89^Zr-Pertuzumab	^177^Lu-Pertuzumab	PET/CT	HER2-positive breast cancer	Phase I	Non-invasive HER2 quantification		[91]
HER2	^64^Cu-Trastuzumab	^177^Lu-Trastuzumab	PET/CT	Breast and gastric cancer	Phase I	Companion diagnostic for HER2-targeted TRT		[56]
DLL3	^89^Zr-DLL3 antibody	^177^Lu-DLL3 antibody	PET/CT	Small-cell lung cancer	Phase I	Highly selective neuroendocrine target		[91]
STEAP1	^89^Zr-DFO-MSTP2109A	^177^Lu-MSTP2109A	PET/CT	Metastatic prostate cancer	Phase I	Highly expressed in advanced prostate cancer		[96]
CAIX	^89^Zr-Girentuximab	^177^Lu-Girentuximab	PET/CT	Clear-cell renal cell carcinoma	Phase I/II	Highly specific renal cancer biomarker		[91]
Mesothelin	^89^Zr-Anti-mesothelin antibody	^177^Lu-Anti-mesothelin	PET/CT	Mesothelioma, ovarian, pancreatic cancer	Phase I	Broad epithelial tumor applicability		
PD-L1	^89^Zr-Atezolizumab	^177^Lu-Anti-PD-L1	PET/CT	Multiple solid tumors	Phase I	Imaging-guided immunotheranostics		[56]
EGFR	^89^Zr-Cetuximab	^177^Lu-Cetuximab	PET/CT	Head & neck, colorectal cancer	Phase I	Enables personalized EGFR-targeted radionuclide therapy		[91]
CD38	^89^Zr-Daratumumab	^177^Lu-Daratumumab	PET/CT	Multiple myeloma	Phase I	Imaging-guided plasma-cell targeting		
CXCR4	^68^Ga-Pentixafor	^225^Ac-Pentixather	PET/CT	Relapsed hematologic malignancies	Phase I	Alpha-emitter for resistant disease		
(D) Emerging Preclinical Next-Generation Theranostic Imaging Agents								
**Molecular Target**	**Diagnostic Imaging Agent**	**Therapeutic Agent**	**Imaging Modality**	**Primary Disease(s)**	**Innovation**	**Preclinical Status**		**Refs.**
LAT1 (L-type amino acid transporter-1)	^18^F-LAT1 tracer	^177^Lu-LAT1 ligand	PET	Glioma, pancreatic cancer	Amino acid transporter theranostics	Preclinical		[91]
Glypican-1 (GPC-1)	^89^Zr-anti-GPC1 antibody	^177^Lu-GPC1 antibody	PET	Pancreatic, breast, ovarian cancer	Highly tumor-specific glycoprotein	Preclinical		
EphA2	^64^Cu-EphA2 peptide	^177^Lu-EphA2 ligand	PET	Glioblastoma, lung, breast cancer	Receptor tyrosine kinase targeting	Preclinical		
TROP-2	^68^Ga-MY6349	^177^Lu-TROP-2 antibody	PET	Triple-negative breast, NSCLC, pancreatic cancer	Companion imaging for ADCs	Preclinical		
Nectin-4	^68^Ga-N188	^177^Lu-Nectin-4 antibody	PET	Urothelial carcinoma, TNBC	Companion imaging for enfortumab therapy	Preclinical/Early translation		[97]
DLL3	^89^Zr-DLL3 antibody	^177^Lu-DLL3 antibody	PET	Small-cell lung cancer	Neuroendocrine tumor targeting	Preclinical		[98,99]
Keratin-1	^68^Ga-KRT1 peptide	^177^Lu-KRT1 ligand	PET	Solid tumors	Novel cell-surface biomarker	Preclinical		[100]
FAP dimer	^68^Ga-DOTAGA.Glu.(FAPI)_2_	^177^Lu-DOTAGA.Glu.(FAPI)_2_	PET	Pan-cancer	Improved tumor retention	Preclinical		[101]
FAP dimer	^68^Ga-DO3A.Glu.(FAPI)_2_	^161^Tb-DO3A.Glu.(FAPI)_2_	PET	Solid tumors	Terbium-based radionuclide therapy	Preclinical		[100]
Integrin αvβ3/FAP dual target	^68^Ga-FAPI-RGD	^177^Lu-FAPI-RGD	PET	Pan-cancer	Dual receptor targeting	Preclinical		[101]
PD-L1	^89^Zr-anti-PD-L1	^177^Lu-anti-PD-L1	PET	Immunotherapy-responsive tumors	Immunotheranostics	Preclinical		[91]
CD47	^89^Zr-CD47 antibody	^177^Lu-CD47 antibody	PET	Hematologic and solid malignancies	Macrophage checkpoint targeting	Preclinical		[102]
Mesothelin	^89^Zr-Mesothelin antibody	^177^Lu-Mesothelin antibody	PET	Mesothelioma, pancreatic, ovarian cancer	Stromal-directed therapy	Preclinical		[91]
Claudin 18.2	^89^Zr-Claudin18.2 antibody	^177^Lu-Claudin18.2 antibody	PET	Gastric, pancreatic cancer	Gastrointestinal precision targeting	Preclinical		[102]
Exosome biomarkers	Radiolabeled exosomes	Drug-loaded engineered exosomes	PET/MRI	Multiple cancers	Biomimetic nanotheranostics	Preclinical		[103]
Engineered CAR-T cells	Reporter gene PET imaging	Therapeutic CAR-T cells	PET	Hematologic malignancies	Cell-based theranostics	Preclinical		[102]
CRISPR/Cas platforms	CRISPR imaging probes	CRISPR-mediated gene editing	Optical/PET	Genetic disorders, cancer	Genome-editing theranostics	Preclinical		
DNA origami nanostructures	Fluorescent/PET nanoprobes	Programmable drug-loaded DNA nanostructures	PET/Optical	Solid tumors	DNA nanotheranostics	Preclinical		
mRNA/LNP platforms	Radiolabeled lipid nanoparticles	Therapeutic mRNA	PET	Cancer, infectious diseases	RNA theranostics	Preclinical		
Synthetic bacteria/microbial vectors	Bioluminescent/PET reporter systems	Engineered microbial therapeutics	PET/Optical	Hypoxic tumors	Living theranostic medicines	Preclinical		[103]

Abbreviations: ADCs, antibody–drug conjugates; CD20, cluster of differentiation 20; EMA, European Medicines Agency; ER, estrogen receptor; FDA, Food and Drug Administration; GEP-NETs, gastroenteropancreatic neuroendocrine tumors; GPC-1, glypican-1; HDE, Humanitarian Device Exemption; KRT1, keratin 1; MDP, methylene diphosphonate; MIBG, metaiodobenzylguanidine; mCRPC, metastatic castration-resistant prostate cancer; NET, norepinephrine transporter; NIS, sodium-iodide symporter; NSCLC, non-small cell lung cancer; PPGL, pheochromocytoma and paraganglioma; PRRT, peptide receptor radionuclide therapy; TNBC, triple-negative breast cancer.

**Table 4:** Disease-specific molecular targets and imaging biomarkers for theranostic applications.

Disease	Molecular Target/Biomarker	Imaging Biomarker/Radiotracer	Imaging Modality	Therapeutic Application	Clinical Status	Refs.
Prostate cancer	PSMA	^68^Ga-PSMA-11, 18F-DCFPyL	PET/CT	177Lu/225Ac-PSMA radioligand therapy	FDA-approved	[91]
	GRPR	^68^Ga-RM2, NeoBOMB1	PET	GRPR-targeted radionuclide therapy	Phase I/II	
	STEAP1	89Zr-DFO-MSTP2109A	PET	Antibody-based radionuclide therapy	Early clinical	
Neuroendocrine tumors	Somatostatin receptor-2 (SSTR2)	^68^Ga/64Cu-DOTATATE	PET/CT	177Lu-DOTATATE PRRT	FDA-approved	[117]
	SSTR antagonist	^68^Ga-DOTA-JR11	PET	177Lu-DOTA-JR11	Phase III	
Breast cancer	HER2	^89^Zr-Trastuzumab	PET	HER2-targeted radionuclide therapy	Phase II	[91]
	Estrogen receptor (ER)	^18^F-FES	PET	Endocrine therapy selection	FDA-approved imaging	
	TROP2	Radiolabeled anti-TROP2 antibodies	PET	TROP2-targeted theranostics	Preclinical	
Lung cancer	EGFR	^89^Zr-Cetuximab	PET	EGFR-targeted radionuclide therapy	Early clinical	
	DLL3	^89^Zr-DLL3 antibody	PET	DLL3-targeted therapy	Phase I/II	
	PD-L1	^89^Zr-Atezolizumab	PET	Immunotheranostics	Early clinical	
Pancreatic cancer	FAP	^68^Ga-FAPI	PET/CT	177Lu-FAPI therapy	Phase II	[118]
	Mesothelin	^89^Zr-Mesothelin antibody	PET	Mesothelin-targeted radionuclide therapy	Early clinical	[91,104]
	GPC-1	Anti-GPC1 PET probe	PET	Targeted nanotheranostics	Preclinical	
Glioblastoma/Glioma	Integrin αvβ3	^68^Ga-RGD	PET	Anti-angiogenic theranostics	Early clinical	
	LAT1	Amino acid PET tracers	PET	LAT1-targeted therapy	Preclinical	
	EphA2	EphA2-targeted PET	PET	Receptor-targeted therapy	Preclinical	
Renal cell carcinoma	CAIX	^89^Zr-Girentuximab	PET	Girentuximab radiotheranostics	Phase III	[95,99]
Hepatocellular carcinoma	Glypican-3 (GPC3)	Anti-GPC3 PET probe	PET	Antibody radionuclide therapy	Early clinical	
Thyroid cancer	Sodium-iodide symporter (NIS)	^123^I/124I	SPECT/PET	131I therapy	Standard clinical practice	[93,104]
Lymphoma	CD20	^111^In-Ibritumomab	SPECT	90Y-Ibritumomab	FDA-approved	
Multiple myeloma	CXCR4	^68^Ga-Pentixafor	PET	Pentixather therapy	Phase II	[85,91]
	CD38	89Zr-Daratumumab	PET	CD38-targeted therapy	Early clinical	
Melanoma	MC1R	α-MSH analogs	PET	Melanoma-targeted radionuclide therapy	Preclinical	
Alzheimer’s disease	Amyloid-β	^18^F-Florbetapir, 18F-Florbetaben	PET	Patient selection for anti-amyloid therapy	FDA-approved imaging	[79,89]
	Tau	^18^F-Flortaucipir	PET	Tau-targeted therapeutic monitoring	FDA-approved imaging	
	Neuroinflammation (TSPO)	TSPO PET tracers	PET	Anti-inflammatory therapy monitoring	Clinical research	
Parkinson’s disease	Dopamine transporter (DAT)	^123^I-Ioflupane	SPECT	Disease diagnosis and progression	FDA-approved	
Atherosclerosis	VCAM-1	VCAM-targeted PET	PET	Plaque imaging and targeted therapy	Preclinical	
Cardiovascular disease	Activated macrophages	^68^Ga-DOTATATE	PET	Inflammation-guided therapy	Early clinical	
Myocardial fibrosis	FAP	^68^Ga-FAPI	PET	Cardiac remodeling assessment	Early clinical	
Autoimmune diseases	Fibroblast activation protein	^68^Ga-FAPI	PET	Fibrosis-targeted therapy	Early clinical	
	Integrins	RGD PET tracers	PET	Immune-cell imaging	Preclinical	
Infectious diseases	Bacterial metabolism	^18^F-FDG, pathogen-specific tracers	PET	Infection localization and treatment monitoring	Clinical	
	Immune-cell activation	TSPO PET tracers	PET	Host inflammatory response monitoring	Clinical research	

**Table 5:** Artificial intelligence applications in theranostic imaging: current evidence, clinical utility, and translational challenges.

AI Domain	AI Technique	Input Data	Primary Application in Theranostics	Representative Disease	Potential Clinical Utility	Evidence Status	Key Translational Challenges	Refs.
Radiomics	Handcrafted quantitative feature extraction	PET/CT, PET/MRI, MRI, SPECT	Quantitative characterization of tumor heterogeneity, target expression and treatment response features	Prostate, neuroendocrine, lung and other cancers	May provide complementary quantitative biomarkers for patient stratification and response assessment	Early clinical/investigational	Feature instability, scanner/protocol dependence, segmentation variability, limited external validation and clinical-utility evidence	[126,127]
Deep radiomics	CNN/deep feature learning	PET, PET/CT, MRI	Automated representation learning and quantitative phenotyping	Oncology	May reduce dependence on handcrafted features and capture complex imaging patterns	Early clinical/investigational	Large representative datasets, interpretability, domain shift, reproducibility and external validation	[126,127]
Deep learning for imaging	CNNs, U-Net, vision transformers	PET/CT, MRI, SPECT/CT	Lesion detection, segmentation, image reconstruction and quantitative image analysis	Cancer, neurological and cardiovascular disorders	Reduces manual workload and may improve consistency of image-derived measurements	Clinical implementation in selected tasks	Generalizability across institutions, scanner/protocol variation, calibration, human benchmarking and prospective validation	[127,128]
Machine learning	Random forest, SVM, XGBoost and related models	Imaging + clinical + laboratory data	Patient stratification and prediction of diagnostic or therapeutic outcomes	Oncology	Integrates heterogeneous predictors to support individualized risk or treatment assessment	Early clinical	Dataset heterogeneity, missing data, class imbalance, model calibration and clinical interpretability	[128]
Therapy-response prediction	Ensemble learning/supervised ML	PET/CT or MRI + clinical variables	Prediction of response or progression following radiopharmaceutical or systemic therapy	Prostate cancer, neuroendocrine tumors	Could support identification of patients less likely to benefit from a planned therapy	Investigational/early clinical	Retrospective datasets, endpoint heterogeneity, limited prospective validation and uncertainty in treatment-effect prediction	[127,128]
Radiogenomics	Multimodal ML/deep learning	PET/CT or MRI + genomic data	Association of imaging phenotypes with molecular alterations	Glioma, breast and lung cancer	Non-invasive molecular characterization and potential patient stratification	Early clinical/investigational	Limited paired datasets, biological heterogeneity, reproducibility and validation across populations	[127]
Imaging genomics/multimodal AI	Deep multimodal learning	PET/MRI + transcriptomics/genomics	Molecular subtype and therapeutic vulnerability prediction	Precision oncology	May connect imaging phenotype with molecular information without repeated invasive sampling	Emerging	Small multimodal cohorts, missing modalities, high dimensionality, data harmonization and external validation	[127]
Digital pathology integration	Whole-slide image AI + multimodal learning	Histopathology + PET/CT/MRI	Integration of tissue morphology with imaging phenotypes and biomarker information	Cancer	May strengthen molecular target characterization and cross-modal biomarker validation	Early clinical/investigational	Annotation quality, inter-institutional pathology variation, multimodal data alignment and clinical validation	[127]
Multiomics integration	Graph neural networks/multimodal AI	Imaging + genomics + transcriptomics + proteomics/metabolomics	Systems-level disease characterization and patient stratification	Precision oncology	Enables integrated biological profiling and hypothesis generation for individualized therapy	Emerging	High computational complexity, dimensionality, missing data, biological validation and reproducibility	[127]
Foundation AI models	Vision–language and multimodal foundation models	PET, CT, MRI, pathology, EHR	Cross-modal image interpretation, information synthesis and decision support	Multiple diseases	Potentially supports scalable multimodal analysis and transfer across tasks	Emerging	Hallucination, domain shift, dataset bias, reproducibility, regulatory qualification and clinical safety	[128]
Explainable AI (XAI)	SHAP, Grad-CAM, attention maps and attribution methods	AI model outputs + imaging/clinical data	Explanation and visualization of model-derived predictions	Oncology and imaging applications	May improve transparency, error analysis and clinician–AI interaction	Emerging/supportive technology	Explanations may not represent causal reasoning; lack of standardized evaluation and uncertain effect on clinical decisions	[127]
Generative AI	Diffusion models, GANs and multimodal generative models	Multimodal imaging	Image reconstruction/enhancement, data augmentation and synthetic-data generation	Oncology and medical imaging	May improve image quality or address limited training-data availability	Emerging	Hallucinated or anatomically implausible structures, synthetic-data bias, validation of generated images and regulatory concerns	[127,129]
Adaptive therapy planning	Reinforcement learning/sequential decision models	Serial PET/CT, treatment and clinical-response data	Dynamic treatment adaptation according to longitudinal response	Radiotheranostics	Provides a framework for response-adaptive treatment strategies	Experimental/preclinical–early clinical	Limited prospective evidence, safety constraints, reward-function design, uncertainty and human oversight	[128]
AI-assisted dosimetry	Regression, deep learning and hybrid AI models	PET/SPECT quantitative imaging, serial scans	Organ/tumor segmentation, activity estimation and patient-specific absorbed-dose prediction	PSMA- and SSTR-targeted radiopharmaceutical therapy	May reduce dosimetry workload and facilitate individualized dose estimation	Early clinical/investigational	Accurate quantitative imaging, limited training data, radionuclide kinetics, dose-model uncertainty and protocol standardization	[128,129]
Theranostic digital twins	Mechanistic/physics-informed models + AI	PET/SPECT + pharmacokinetic, genomic and clinical data	Simulation of radiopharmaceutical distribution, absorbed dose and treatment response	Nuclear oncology	May support patient-specific treatment simulation and model-informed therapy planning	Preclinical–early clinical	Verification/validation, uncertainty quantification, data integration, computational burden and lack of prospective clinical validation	[130,131]
Digital twins	Physics-informed AI + computational modeling	Imaging + clinical + longitudinal data	Dynamic patient-specific disease and treatment simulation	Oncology, cardiovascular disease	Potentially enables longitudinal treatment simulation and individualized decision support	Emerging	Definitional inconsistency, high computational demands, incomplete data, external validation and clinical benchmarking	[131]
Autonomous imaging/workflow AI	Workflow automation and AI quality-control models	PET/CT, MRI, SPECT/CT	Image acquisition assistance, quality assessment and workflow optimization	Multiple imaging specialties	May reduce operator variability and improve workflow efficiency	Emerging	Hardware/software interoperability, robustness to unusual cases, regulatory approval and human oversight	[127]
Clinical decision support	Hybrid multimodal AI	Imaging + EHR + pathology + laboratory data	Integrated patient stratification and treatment-support recommendations	Precision oncology	Can synthesize heterogeneous information for multidisciplinary decision support	Early clinical	Interpretability, interoperability, calibration, responsibility for decisions and prospective clinical-impact assessment	[132]
Real-time therapy monitoring	Longitudinal/sequential AI	Serial PET/CT or PET/MRI + clinical data	Early assessment of treatment response and identification of non-responders	Oncology	May enable earlier treatment reassessment than conventional endpoint evaluation	Early clinical/investigational	Temporal data variability, standardized response definitions, prospective multicenter validation and demonstrated outcome benefit	[126]

**Table 6:** Regulatory, manufacturing, and translational challenges in theranostic imaging agents.

Challenge Domain	Key Challenge	Scientific/Technical Issue	Clinical Impact	Current Mitigation Strategies	Future Direction	Refs.
Regulatory approval	Fragmented global regulatory pathways	Different FDA, EMA, PMDA and national requirements	Delayed multinational approvals	Harmonized regulatory guidance	International regulatory convergence	[138,139]
Companion diagnostics	Co-development of imaging and therapeutic agents	Separate approval pathways for diagnostic and therapeutic components	Delays in clinical translation	Parallel clinical development	Unified theranostic regulatory framework	[139,140]
Radiopharmaceutical manufacturing	Short radionuclide half-life	Time-sensitive synthesis, QC and distribution	Limited patient access	Decentralized production networks	Automated regional manufacturing hubs	[138,141]
GMP compliance	Complex GMP requirements	Sterility, radionuclidic purity, batch consistency	Increased production costs	Automated synthesis modules	Continuous GMP manufacturing	[141]
Quality control	Multi-parameter release testing	Radiochemical purity, stability, endotoxin testing	Delayed product release	Rapid analytical assays	AI-enabled real-time QC	[138,142]
Radionuclide supply	Limited isotope availability	Global shortages of 177Lu, 225Ac, 64Cu and emerging radionuclides	Treatment delays	Expanded production capacity	Cyclotron and accelerator-based production	[138,142]
Chelator chemistry	Variable in vivo stability	Radionuclide dissociation and off-target uptake	Reduced therapeutic index	Improved bifunctional chelators	Universal chelation platforms	[143]
Scale-up manufacturing	Difficult laboratory-to-industrial transition	Complex synthesis and reproducibility	Commercialization bottleneck	Process analytical technologies (PAT)	Modular automated manufacturing	[138,143]
Personalized dosimetry	Lack of standardized dosimetry protocols	Variable absorbed-dose estimation	Risk of under- or over-treatment	Voxel-based dosimetry	AI-assisted personalized dosimetry	[144]
Safety and toxicology	Off-target radiation exposure	Bone marrow, kidneys and salivary glands at risk	Dose-limiting toxicity	Patient-specific dose optimization	Predictive toxicity modeling	[139]
Clinical validation	Limited multicenter randomized trials	Small patient cohorts	Restricted evidence generation	International collaborative trials	Global prospective registries	[14]
Standardization	Lack of harmonized imaging protocols	Scanner and reconstruction variability	Poor reproducibility	EANM/SNMMI harmonization	International imaging standards	
Biomarker validation	Variable target expression	Biological heterogeneity	Inconsistent patient selection	Companion diagnostic imaging	Multiomics biomarker integration	
Artificial intelligence	Lack of validated AI algorithms	Limited external validation and transparency	Reduced clinical trust	Explainable AI (XAI)	Regulatory-qualified AI models	[142]
Data interoperability	Disparate imaging and clinical databases	Poor integration across institutions	Inefficient precision medicine workflows	FAIR data principles	Federated learning platforms	[142]
Economic considerations	High development and treatment costs	Expensive radionuclide production and infrastructure	Limited reimbursement	Health technology assessment	Value-based reimbursement models	[142,143,144]
Infrastructure	Limited theranostic centers	Specialized equipment and workforce required	Geographic disparities	Regional referral networks	Global theranostic centers of excellence	[138]
Workforce training	Shortage of multidisciplinary expertise	Need for nuclear medicine, radiochemistry, physics and oncology integration	Slower adoption	Specialized educational programs	AI-assisted clinical workflows	[143]
Ethical considerations	Radiation safety, informed consent and AI governance	Privacy, algorithmic bias and equitable treatment access	Ethical implementation challenges	Transparent governance frameworks	Responsible AI and ethical theranostics	[142]
Health equity	Unequal global access	Infrastructure and radionuclide supply disparities	Limited availability in low-resource settings	International collaborations	Affordable decentralized production	[43,144]
Translational gap	Slow bench-to-bedside conversion	Complex regulatory, manufacturing and clinical requirements	Delayed patient benefit	Translational research networks	Integrated precision medicine ecosystem	[138,143]
Future readiness	Integration of AI, digital twins and adaptive therapy	Need for regulatory frameworks for computational medicine	Emerging implementation challenges	Pilot clinical studies	AI-driven, continuously learning theranostic platforms	[143,144]

## Data Availability

No new data were created or analyzed in this study. Data sharing is not applicable to this article.

## Abbreviations

The following abbreviations are used in this manuscript:

AI	Artificial Intelligence
AI/ML	Artificial Intelligence/Machine Learning
CT	Computed Tomography
DL	Deep Learning
EHR	Electronic Health Record
FAP	Fibroblast Activation Protein
FAPI	Fibroblast Activation Protein Inhibitor
ICD	International Classification of Diseases
LLM	Large Language Model
MRI	Magnetic Resonance Imaging
MOF	Metal–Organic Framework
NIR	Near-Infrared
PET	Positron Emission Tomography
PSMA	Prostate-Specific Membrane Antigen
PSMA-PET	Prostate-Specific Membrane Antigen Positron Emission Tomography
PSMA-617	Prostate-Specific Membrane Antigen-617
ROS	Reactive Oxygen Species
SPECT	Single-Photon Emission Computed Tomography
CCK2R	Cholecystokinin 2 Receptor
CXCR4	C-X-C Chemokine Receptor Type 4
DOTATATE	DOTA-(Tyr3)-Octreotate
FAPI-PET	Fibroblast Activation Protein Inhibitor Positron Emission Tomography
GRPR	Gastrin-Releasing Peptide Receptor
PET/CT	Positron Emission Tomography/Computed Tomography
PET/MRI	Positron Emission Tomography/Magnetic Resonance Imaging
SSTR	Somatostatin Receptor
TAT	Targeted Alpha Therapy
TRT	Targeted Radionuclide Therapy
RLT	Radioligand Therapy
RPT	Radiopharmaceutical Therapy
SIRT	Selective Internal Radiation Therapy
TARE	Transarterial Radioembolization
TCR	T-cell Receptor
CAR-T	Chimeric Antigen Receptor T-cell
CD	Cluster of Differentiation
EGFR	Epidermal Growth Factor Receptor
HER2	Human Epidermal Growth Factor Receptor 2
PD-L1	Programmed Death-Ligand 1
PD-1	Programmed Cell Death Protein 1
VEGF	Vascular Endothelial Growth Factor
IONPs	Iron Oxide Nanoparticles
AuNPs	Gold Nanoparticles
MOFs	Metal–Organic Frameworks
SPIONs	Superparamagnetic Iron Oxide Nanoparticles
QDs	Quantum Dots
CNN	Convolutional Neural Network
DNN	Deep Neural Network
NLP	Natural Language Processing
CDSS	Clinical Decision Support System
DICOM	Digital Imaging and Communications in Medicine
PACS	Picture Archiving and Communication System
FDG	Fluorodeoxyglucose
Gy	Gray
Bq	Becquerel
MBq	Megabecquerel
GBq	Gigabecquerel
SUV	Standardized Uptake Value
TBR	Tumor-to-Background Ratio
ROI	Region of Interest
VOI	Volume of Interest
TAC	Time–Activity Curve
BED	Biologically Effective Dose
OAR	Organ at Risk
TNBC	Triple-Negative Breast Cancer
